# Activation of Glutamate Transport Increases Arteriole Diameter *in vivo*: Implications for Neurovascular Coupling

**DOI:** 10.3389/fncel.2022.831061

**Published:** 2022-03-04

**Authors:** Joshua G. Jackson, Elizabeth Krizman, Hajime Takano, Meredith Lee, Grace H. Choi, Mary E. Putt, Michael B. Robinson

**Affiliations:** ^1^Children’s Hospital of Philadelphia, Philadelphia, PA, United States; ^2^Department of Pediatrics, University of Pennsylvania, Philadelphia, PA, United States; ^3^Department of Neurology, University of Pennsylvania, Philadelphia, PA, United States; ^4^Department of Biostatistics, Epidemiology & Informatics, University of Pennsylvania, Philadelphia, PA, United States; ^5^Department of Systems Pharmacology and Translational Therapeutics, University of Pennsylvania, Philadelphia, PA, United States

**Keywords:** glutamate transport, arteriole diameter, neurovascular coupling, astrocyte, *in vivo* microscopy

## Abstract

In order to meet the energetic demands of cell-to-cell signaling, increases in local neuronal signaling are matched by a coordinated increase in local blood flow, termed neurovascular coupling. Multiple different signals from neurons, astrocytes, and pericytes contribute to this control of blood flow. Previously, several groups demonstrated that inhibition/ablation of glutamate transporters attenuates the neurovascular response. However, it was not determined if glutamate transporter activation was sufficient to increase blood flow. Here, we used multiphoton imaging to monitor the diameter of fluorescently labeled cortical arterioles in anesthetized C57/B6J mice. We delivered vehicle, glutamate transporter substrates, or a combination of a glutamate transporter substrate with various pharmacologic agents via a glass micropipette while simultaneously visualizing changes in arteriole diameter. We developed a novel image analysis method to automate the measurement of arteriole diameter in these time-lapse analyses. Using this workflow, we first conducted pilot experiments in which we focally applied L-glutamate, D-aspartate, or L-*threo*-hydroxyaspartate (L-THA) and measured arteriole responses as proof of concept. We subsequently applied the selective glutamate transport substrate L-THA (applied at concentrations that do not activate glutamate receptors). We found that L-THA evoked a significantly larger dilation than that observed with focal saline application. This response was blocked by co-application of the potent glutamate transport inhibitor, L-(2S,3S)-3-[3-[4-(trifluoromethyl)-benzoylamino]benzyloxy]-aspartate (TFB-TBOA). Conversely, we were unable to demonstrate a reduction of this effect through co-application of a cocktail of glutamate and GABA receptor antagonists. These studies provide the first direct evidence that activation of glutamate transport is sufficient to increase arteriole diameter. We explored potential downstream mechanisms mediating this transporter-mediated dilation by using a Ca^2+^ chelator or inhibitors of reversed-mode Na^+^/Ca^2+^ exchange, nitric oxide synthetase, or cyclo-oxygenase. The estimated effects and confidence intervals suggested some form of inhibition for a number of these inhibitors. Limitations to our study design prevented definitive conclusions with respect to these downstream inhibitors; these limitations are discussed along with possible next steps. Understanding the mechanisms that control blood flow are important because changes in blood flow/energy supply are implicated in several neurodegenerative disorders and are used as a surrogate measure of neuronal activity in widely used techniques such as functional magnetic resonance imaging (fMRI).

## Introduction

The adult human brain represents about 2% of total body weight, but consumes up to 20% of the basal metabolic rate (for reviews, see Shulman et al., 2004; Hertz et al., 2007; Harris et al., 2012; Stobart and Anderson, 2013; Weber and Barros, 2015). Compared to other organs, the brain has relatively low levels of stored glucose in the form of glycogen (Obel et al., 2012) and instead depends on local delivery of energy substrates by a dense network of blood vessels (Bohn et al., 2016). Acute decreases in and/or a loss of blood flow, such as those observed with stroke or a heart attack, are associated with rapid brain damage (Hinzman et al., 2014; Jackman and Iadecola, 2015), and chronic decreases in blood flow have been observed in individuals with cognitive decline and dementia (Leeuwis et al., 2017, 2018).

The brain matches increases in local metabolic demand caused by neuronal activity with corresponding increases in local blood flow, in a process termed neurovascular coupling (NVC). This process supports the energetic costs of cellular signaling, including costs associated with vesicular transmitter packaging/release and membrane repolarization after activation of ligand-/voltage-gated ion channels (Howarth et al., 2012). Neurovascular coupling provides the signal for functional/BOLD magnetic resonance imaging (fMRI) studies. It also underlies some versions of 2-deoxyglucose-based positron emission tomography (PET) imaging, and near-infrared spectroscopy (Raichle and Mintun, 2006; Hillman, 2014).

Release of neurotransmitters, particularly glutamate, stimulates increases in Ca^2+^ in astrocytes and results in the production/release of various vasoactive molecules on to the vasculature to drive changes in vessel diameter (Porter and McCarthy, 1996; Zonta et al., 2003; Filosa et al., 2004; Mishra et al., 2016). Uncaging of Ca^2+^ from astrocyte endfeet is sufficient to evoke vessel dilation (Takano et al., 2006), and inhibition of metabotropic glutamate receptor 5 (mGluR5) reduces neurovascular coupling evoked by odor (Petzold et al., 2008) and whisker stimulation (Wang et al., 2006) *in vivo*. The role of mGluR5 activation in this response is, however, complicated by the observation that most mGluR5 is expressed by neurons in the cortex in the adult nervous system (Sun et al., 2013; Zhang et al., 2014). Interestingly many of these studies also implicated an additional pathway, glutamate uptake, in mediating NVC (Gurden et al., 2006; Petzold et al., 2008; Schummers et al., 2008). Using genetic approaches to reduce expression of astrocytic glutamate transporters, three different groups implicated glutamate uptake in the regulation of NVC (Voutsinos-Porche et al., 2003; Herard et al., 2005; Martin et al., 2012). Three additional studies have also demonstrated that acute inhibition of glutamate uptake with threo-ß-benzyloxyaspartate (TBOA) attenuates stimulus-evoked changes in the intrinsic optical signal (a measure of blood O_2_) or stimulus-induced increases in blood flow in olfactory bulb or visual cortex (Gurden et al., 2006; Petzold et al., 2008; Schummers et al., 2008). Together, these studies suggested that glutamate transport is required for neuronal activity-dependent increases in blood flow, but it has not been determined if activation of glutamate transport is sufficient to increase blood flow.

In this study, we developed an approach pairing multiphoton imaging of arteriole diameter with focal application of glutamate transporter substrates and pharmacological inhibitors in the cortex of mice. We provide direct evidence that activation of glutamate transport is sufficient to cause an increase in arteriole diameter. We show that this effect is blocked by co-application of a selective, pan inhibitor of glutamate transporters, but there was little evidence of inhibition by a cocktail of glutamate and GABA receptor antagonists. We explored the effects of a number of pharmacologic inhibitors of some of the downstream signals that have been implicated in neurovascular coupling. While a clear effect of transporter substrates on arteriole diameter is observed, limitations to the study design prevent us from making firm conclusions as to the ultimate downstream mediators. We discuss these limitations and provide a roadmap for potential next steps.

## Materials and Methods

### Materials

**Table:** 

Reagent	Dose/Concentration (if applicable)	Company	Catalog Number
C57BL/6J mice		Jackson Laboratories	000664
Head Plate		Narishige	CP-1
VetBond		3M	006245
Dental Acrylic		Lang Dental Mfg. Co. Inc.	1334
Trephine drill bit		Fine Science Tools	18004-27
Borosilicate Glass Capillaries	Used to make glass pipettes	Kwik-Fil	18150F-4
Isofluorane	3–4% induction, 1-2% maintenance	Piramal	66794-017-10
Alpha Chloralose	5mg/ml in 20% PEG. Inject 50 ug/g mouse weight	Sigma	23120
PEG; Polyethylene glycol	20% solution in saline	Fluka	81300
Fluorescein Dextran, 70,000 MW, Anionic	5% stock solution in saline. Inject 65 μl in tail vein.	Invitrogen	D1823
Rhodamine Dextran, 10,000 MW, Anionic	5% stock solution in saline. Dilute to 0.5% with drug/vehicle for picospritz.	Invitrogen	D1824
AlexaFluor 633 hydrazide	1mg/ml stock solution in saline. Inject 25 μl in tail vein.	Invitrogen	A30634
L-Glutamic acid	100 μM and 1mM in ACSF. Equimolar equivalent of NaOH. pH = 7.4	Sigma	G1251
D-Aspartic acid	100 μM and 1 mM in ACSF. Equimolar equivalent of NaOH. pH = 7.4	Sigma	219096
L-THA; L-(-)-threo-3-Hydroxyaspartic acid	100 μM and 1 mM in ACSF Equimolar equivalent of NaOH. pH = 7.4	Tocris	0183
[1.2pt] TFB-TBOA; (3S)-3-[[3-[[4-(Trifluoromethyl) benzoyl]amino]phenyl] methoxy]-L-aspartic acid	1 μM in ACSF Equimolar equivalent of NaOH, pH = 7.4	Tocris	2532
KB-R7943; 2-[2-[4-(4-Nitrobenzyloxy)phenyl]ethyl] isothiourea mesylate	15 μM in 0.1% DMSO in ACSF	Tocris	1244
[1.2pt] YM-244769; N-[(3-Aminophenyl)methyl]-6-[4-[(3-fluorophenyl)methoxy] phenoxy]-3-pyridinecarboxamide dihydrochloride	1 μM in 0.1% DMSO in ACSF	Tocris	4544
DNQX; 6,7-Dinitroquinoxaline-2,3-dione	10 μM in ACSF	Tocris	2312
D-APV; D-(-)-2-Amino-5-phosphonopentanoic acid	50 μM in ACSF	Tocris	0106
MCPG; (RS)-α-Methyl-4-carboxyphenylglycine disodium salt	1 mM in ACSF	Tocris	3696
R-Baclofen; (R)-4-Amino-3-(4-chlorophenyl)butanoic acid	100 μM in ACSF	Tocris	0796
BAPTA; 1,2-Bis(2-aminophenoxy)ethane-N,N,N′,N′-tetraacetic acid tetrakis(acetoxymethyl ester)	200 μM in ACSF	Tocris	2787
NPA; N5-[Imino(propylamino)methyl]-L-ornithine hydrochloride	2 μM in ACSF	Tocris	1200
Indomethacin; 1-(4-Chlorobenzoyl)-5-methoxy-2-methyl-1H-indole	10 μM in 0.1% DMSO in ACSF	Tocris	1708
Dimethyl sulfoxide (DMSO)		Sigma	34869

### Source of Mice and Preparation for Imaging

This study was reviewed and approved by the Institutional Animal Care and Use Committee at The Children’s Hospital of Philadelphia prior to performing these procedures and imaging. Adult (6–12 weeks old) male and female C57BL/6J mice were initially purchased from an outside vendor and used to establish a breeding colony that was maintained at the Children’s Hospital of Philadelphia. Replacement breeders were periodically purchased from the same vendor to prevent genetic drift. If acquired from an outside vendor, the mice acclimated to the animal facility for at least 24 h prior to the procedure. Mice were housed in cages of two to five in 12-h light/dark cycles and given access to food *ad libitum*.

Mice were anesthetized in an induction chamber by inhalation of 3–4% isoflurane. Once the mouse was anesthetized as evidenced by lack of spontaneous movement or vocalization and lack of response to a tail or paw pinch, the mouse was placed on a heated pad and the head was stabilized in a stereotaxic frame (Kopf Instruments). The mouse was kept under 1–2% isofluorane anesthesia throughout the surgical procedure. Alexafluor633 hydrazide (Invitrogen, 25 μl of 1mg/ml in saline) was injected via the lateral tail vein to allow selective labeling of the arteries (Shen et al., 2012). This was done prior to surgery to allow sufficient time for the dye to label the vascular smooth muscle cells, but clear from the vessel lumen. One to two drops of sterile eye drops were applied to each eye prior to surgery to protect from dehydration. The fur on the mouse’s head was removed with scissors and the skin was cleaned with a 70% alcohol wipe. Using surgical scissors an incision was made along the midline. The skull surface was scraped with a scalpel so the VetBond used in the next step could adhere efficiently. A head plate with a 5 mm diameter observation hole was glued (using VetBond) to the skull. The plate was further sealed to the skull with dental acrylic. The acrylic was allowed to set for 20 min prior to drilling. A small craniotomy over the somatosensory cortex (2.4 mm in diameter) was made 1mm posterior to bregma and 3.5 mm lateral from midline over on the right hemisphere (Sato et al., 2007) with a trephining bit using a high-speed surgical drill (Foredom) taking care to avoid penetrating the dura. The circular flap of bone was removed to expose the dura, which was then dissected off the surface of the brain using a pair of fine tip (Dumont #5.5) forceps and a surgical needle (30G) bent to form a hook-like cutting tool. The surface of the cortex was kept moist with sterile artificial cerebrospinal fluid (ACSF; 125 mM NaCl, 5 mM KCl, 10 mM glycine, 10 mM HEPES, 3.1 mM CaCl_2_, 1.2 mM MgCl_2_, 10 mM glucose, pH 7.4, sterile filtered). Extreme care was taken to ensure that the cortical surface was undamaged. The surface was sealed with low-melt agarose (1% in sterile ACSF w/v). A rectangular piece of #1 coverglass was positioned on top of the agarose and secured with a small drop of acrylic dental cement at the edges. A small gap, oriented perpendicular to the micropipette, was left uncovered at the edge of the craniotomy to allow pipette placement. To visualize the vasculature, Fluorescein Dextran (70K MW, Invitrogen, 65 μl of 5% solution in saline w/v) was injected into the tail vein. Others have loaded BAPTA-AM or Ca^2+^ dyes to the cortex which are subsequently dispersed through the astrocytic syncytium (Nimmerjahn et al., 2004). For one experimental group, the Ca^2+^ chelator/indicator, BAPTA-AM (20 μM) was applied to the cortical surface for 20 min followed by a rinse with sterile ACSF and sealing with low-melt agarose as described above. Then Fluorescein Dextran was injected as described above. Next, the mouse was transitioned from isoflurane to alpha chloralose anesthesia (50 μg/g, intraperitoneal) followed by a slow elimination of isoflurane.

### 2-Photon Imaging

Following installation of the cranial window, the entire stereotactic base (SR-9AM, Narishige) with the mouse mounted by a head plate (CP1, Narishige) was placed on the microscope stage (MP200, Thorlabs). Glass micropipettes (borosilicate Kwik-Fil, World Precision Instruments) were prepared on a Flaming-Brown pipette puller (Sutter). Resistance of each pipette (∼10 MΩ) was verified prior to use to prevent leakage of dye/drug into the cortex prior to pressure ejection. Glass micropipettes were filled with vehicle (saline or saline with 0.1% DMSO both containing 0.5% Rhodamine Dextran; Invitrogen) or vehicle with pharmacologic agents. The pipette was attached to a pressure transduction system (Picospritzer III, Parker Hannifin) and was roughly positioned over a field containing a descending arteriole using a micromanipulator (ROE-200, Sutter Instruments) while visualized using a 4x objective under epi-illumination. The objective was then switched to a water immersion 16x lens (LWD, NA = 0.8, Nikon) attached to a Thorlabs Bergamo multiphoton microscope equipped with a femtosecond laser (MaiTaiDeepSee, Spectra Physics), and images were captured with ThorImage software (Thorlabs). An area containing 1-4 arterioles was identified based on the Alexafluor633 hydrazide and the pipette was visualized with rhodamine such that the distance between both was kept relatively consistent at approximately 40 μm. We utilized an episodic imaging framework with each epoch consisting of a 5-sec baseline period followed by a 50 ms (5psi) pressure pulse and a 2-min acquisition period (10–20Hz sampling frequency). Based on the pilot experiments and previous reports in the literature, we anticipated relatively small effect sizes and possible variability in the responses, therefore we recorded a total of 3 pulses from each field with a 5-min recovery period between each pulse. The pulse delay (relative to acquisition start) was controlled using ThorImage software and a Master8 pulse stimulator (AMPI). To further control for pipette leaks, a sham imaging epoch was performed prior to each experiment, where the pipette was positioned close to the vessel, but no pressure injection was initiated. Injection (or leak) were verified by increases in red fluorescence intensity (0.5% Rhodamine Dextran) at the tip of the pipette. In several cases, the effects of a particular agent or vehicle were examined in more than one field, but no more than 2 fields were included in the analysis. At the end of the imaging session, the mouse was euthanized by cervical dislocation while still under anesthesia.

### Image Analyses

Initially, images were analyzed in a semi-automated manner using a series of custom macros in ImageJ/FIJI. Briefly, a user-defined line was placed perpendicular to the long-axis of the arteriole and the profile of the vessel (full-width, half max) calculated for all time-points in the image series. Multiple parameters including max change in vessel diameter, area-under the curve, and vessel duration were extracted from the xt profile. These programs were used to analyze the initial observations of L-glutamate, D-aspartate, and L-THA-evoked changes in vessel diameter.

To standardize the analyses, we subsequently created a fully automated, multi-step workflow to analyze the arteriole images. First, ImageJ/FIJI was used to de-interleave the two channels and standardize all the time-series images (videos). One channel captured Fluorescein Dextran which was used to measure arteriole diameter, and in the other channel Rhodamine Dextran and AlexaFluor 633 fluorescence were used to test for pipette leaks during the initial sham imaging epoch, identify the pulse frame, confirm pressure ejection, and measure the distance between the pipette and the vessel. The videos were standardized according to the following parameters: Scale change to width 544 pixels, height 336 pixels, depth 1400 images, Interpolation Bilinear, Average process; Converted to Gray scale; and saved as 8-bit tiff files. Next, we analyzed the Fluorescein Dextran videos of the arterioles using custom Matlab code. Zonta and colleagues previously drew several lines across each vessel to measure changes in diameter over time (Zonta et al., 2003). We used a similar strategy and the first Matlab program, BloodV10, automatically drew up to 8 lines perpendicular to the centerline of the arteriole of interest. When the program identified more than one useable arteriole, one arteriole was randomly chosen and kymographs from the resulting time-series images were generated. Lines that did not cross the arteriole were eliminated. Artifacts caused by movements during imaging were detected by calculating an image feature related to the focus of the image at each timepoint, e.g., edge sharpness. The focus feature was normalized to baseline and if it exceeded three standard deviations above or below baseline recording, the data at the time point were excluded from subsequent analysis. The second Matlab program, KymoHT6, applied a spatial mean filter and binarized the kymographs using two Gaussian fit to image intensity histogram to yield the width of the arteriole as a function of time. The width data were averaged every second and normalized to baseline (3 s before the pulse). The MATLAB programs created for this analysis are freely available on GitHub^1^.

The data from all kymographs for each line were exported and using the area-under-the-curve analysis function in Graphpad PRISM 9.0, the number of peaks, the total area under each individual peak (AUC), as well as data about each individual peak including the maximum change, the time of this maximum change, and the times of the beginning and end of each peak were calculated for each line profile. Baseline was set at zero and only dilations were quantified. Analyses of these results included some exploratory data visualization as well as data cleaning and transformation. These steps were performed reproducibly using Python code (version 3.8) in Jupyter notebooks.^2^ To visualize the diameter of the blood vessel over time for each pulse, the data for that pulse, including up to 8 line profiles, were isolated using a unique pulse identifier and plotted. To identify the peak with the largest area, the original data were transposed so that the individual line profiles were put in rows, and the peak data were put in columns. From there, the maximum percent area could be identified. Data cleaning to unambiguously identify column names was required. The data for the peak with the largest percent area for each line profile were saved in new data file.

**FIGURE 1:**
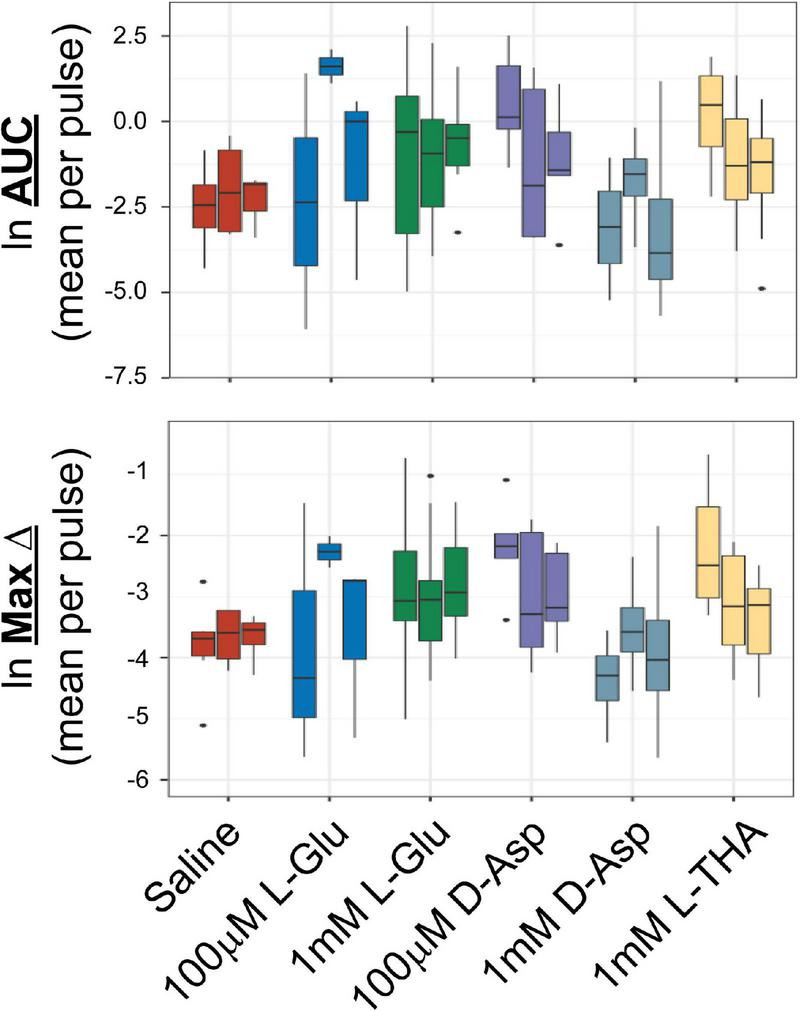
Effects of saline or glutamate transporter substrates on arteriole diameter. Saline, L-glutamate, D-aspartate, or L-THA were picospritzed near cortical arterioles. The area of the largest dilation and the maximum change were measured as described in the text. The original data were the mean values for each animal. The median is indicated by the horizontal line and the interquartile range (25^th^ to 75^th^ percentile) by the box. The whiskers are 1.5 times the interquartile range, added or subtracted to the percentiles, and drawn back to the nearest point. Values outside of the whiskers are outliers (dots). The data for saline are from 6 animals (3 male,3 female), the data for 100 μM L-Glu are from 3 animals (1 male, 2 females), the data for 1 mM L-Glu are from 12 animals (12 males), the data for 100 μM D-Asp are from 5 animals (3 males, 2 females), the data for 1 mM D-Asp are from 4 animals (4 males), the data for 1 mM L-THA are from 11 animals (7 males, 4 females).

The numbers of animals used in preliminary studies are presented in the legend to Figure 1. For the rest of the studies, a total of 99 animals (48 males, 51 females) were initially used with 93 (94%) yielding usable data. The number of animals per experimental group ranged from 3 (receptor antagonists) to 12 (100 μM L-THA alone) (see figure legends for specific numbers). Data were excluded based on the following criteria: BloodV10 was unable to draw lines across the vessel, KymoHT6 could not analyze at least 5 kymographs/lines for an individual pulse, no positive peaks were identified, gaps in recordings were greater than 20 s, fewer than 60 s of data out of 120 s, or maximum change was greater than 30%. The final analytic dataset included a total of 3055 lines.

### Statistical Analyses

Initially, to explore the distribution of the area of the largest arteriole dilation (AUC) and maximum change in diameter variables, as well as broad trends across groups, we created boxplots using data pooled across fields and animals. The boxplots indicated AUC and maximum change, were strongly right-skewed [positive]. To fit the statistical models, the data were log-transformed (Ln) to achieve approximate normality of the independent variable. We fit a mixed effects model using either AUC or maximum change as the dependent variable with a random intercept term, allowing for correlations between repeated measurements on the same animal and indicating individual animals as the independent unit of analysis. Pharmacologic agent, field, pulse and the interaction between pulse and pharmacologic agent were included as fixed independent variables. A single model was fit to the entire dataset in order to use both the same negative controls (saline alone or saline plus DMSO) and positive intervention (L-THA alone) throughout. The interaction term potentially allowed the effects of a pharmacologic agent to vary by pulse; because an omnibus likelihood ratio test indicated that interaction term was highly significant (*p* < 0.001 for both AUC and Max Change), this term was retained in the final model. Using the fitted model, we constructed contrasts of interest and then exponentiated to determine ratios of the mean outcome for pairs of groups. For agents dissolved in saline, the contrasts were formed based on a direct comparison with saline, or L-THA in saline. For agents dissolved in Saline/DMSO, we first estimated the effect of the agent alone, or agent in combination with L-THA, by simply subtracting the mean of the Saline/DMSO control. The effect of the agent in combination with L-THA versus L-THA was estimated using a difference of difference approach, i.e., we took the difference of the agent plus L-THA in Saline/DMSO versus the Saline/DMSO control and then subtracted the difference between L-THA in Saline versus Saline alone. In this way we compared effects of the agent with L-THA versus L-THA alone after removing effects of Saline and DMSO, noting that no direct effect of L-THA in Saline with DMSO was measured. Using data from each pulse, differences were created on the natural log scale and then exponentiated to obtain a ratio. Effects are reported both for individual pulses, as well as by averaging across pulses. Hypothesis tests were based on Wald tests using a small-sample Satterthwaite correction to the degrees of freedom (Luke, 2017) and were two-sided without adjustment for multiple comparisons. Because the number of animals, and thus the statistical power to detect a specific effect, differed across interventions, ranking the efficacy of the interventions based on p-values would be misleading (Putt, 2021). Notably p-values lack context without an *a priori* power calculation based on assumptions about effect size and standard deviation. We thus report 95% confidence intervals (CIs) in order to assess and compare possible effect sizes. With high probability, these 95% CIs cover the unknown effect size under the hypothetical scenario that an infinite number of animals could be sampled and analyzed; the width of the CI reflects the precision of the estimate, a function of both the variability in the outcome and the number of animals. Unlike p-values, no *a priori* assumptions about effect sizes, standard deviations or sample size are needed to accurately interpret a 95% CI. Using forest plots, we report both the mean of the three pulses to summarize possible effects, as well as means for the individual pulses. Specifically, we report contrasts between the pharmacologic agents and the appropriate control, exponentiated to yield relative effects (ratios of the intervention to the appropriate control). For L-THA alone and L-THA with TFB-TBOA, the model for AUC was repeated separately for the male and female animals to assess the possibility of effects related to biological sex. Analyses were carried out in R version 4.0.5 (2021-03-31) R Core Team (2021). Using packages lme4 and lmerTest for the mixed effects models, and ggplot2 for graphics.

## Results

**FIGURE 2:**
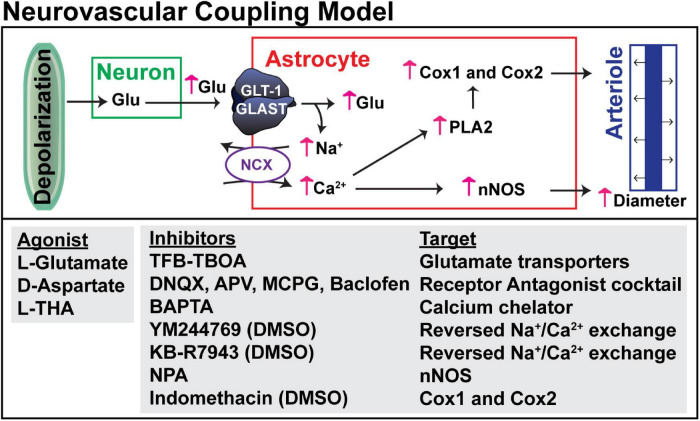
Hypothesized mechanism for glutamate transport-dependent vasodilation. After neuronal depolarization, most of the glutamate released into the extracellular space is cleared by the astrocytic glutamate transporters, GLT-1 and GLAST. Several groups have shown that the simultaneous influx of Na^+^ that drives glutamate uptake can trigger reversed operation of the Na^+^/Ca^2+^ exchangers and increase in cytosolic Ca^2+^. This Ca^2+^ can activate nitric oxide synthetase (NOS), phospholipase 2 (which generates arachidonic acid and can activate cyclooxygenase, Cox1 and Cox2), or other Ca^2+^-activated processes. These pathways have been linked to vasodilation in several studies (for references, see results). The pharmacologic agents used to test each of these targets is also included.

Several groups have demonstrated that pharmacologic inhibition or genetic deletion of glutamate transporters attenuates, or completely blocks, the increase in energy delivery or blood flow that is observed following neuronal activation (Voutsinos-Porche et al., 2003; Gurden et al., 2006; Petzold et al., 2008; Schummers et al., 2008). These studies imply that glutamate transport contributes to the coupling of neuronal activity to increased blood flow. The goal of the present study was to determine if activation of glutamate transport causes an increase in arteriole diameter *in vivo*. The overall model and the general approach is schematically summarized in Figure 2. In the mammalian brain, the bulk of glutamate transport is mediated by two Na^+^-dependent glutamate transporters, called GLT-1 and GLAST (or EAAT2 and EAAT1, respectively) that are enriched in astrocytes (Rothstein et al., 1994; Danbolt et al., 2016).

**FIGURE 3:**
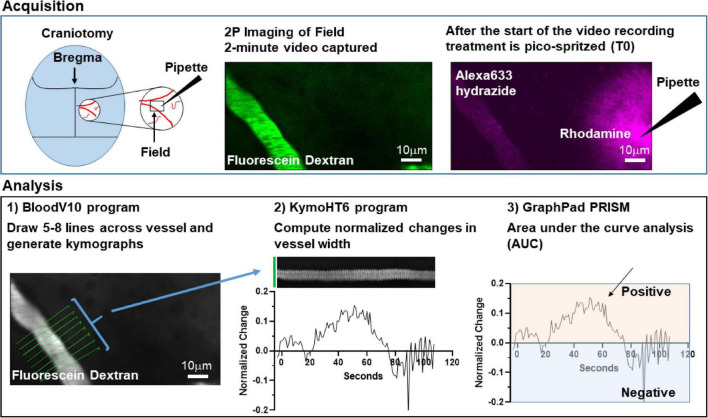
Schematic model of methods used to analyze changes in arteriole diameter.

To determine if activation of glutamate transport is sufficient to cause an increase in arteriole diameter, a small cranial window was made over the primary somatosensory “barrel” cortex in adult mice (Figure 3). Arterioles were differentiated from other blood vessels by injecting AlexaFluor 633 hydrazide (red), a dye that binds to elastin in vascular smooth muscle cells (Shen et al., 2012; Hill et al., 2015), via the lateral tail vein. Fluorescein-dextran (green; 70 kDa) was also injected into the tail vein and used to visualize arteriole diameter before and after a brief pulse of vehicle containing rhodamine-dextran with or without pharmacologic agents. Every application was confirmed by the appearance of extracellular rhodamine fluorescence (see Figure 3). This was repeated two more times at 5 min intervals. In an effort to reduce variability, we imaged arterioles of similar size; the median diameter was 16.6 μm (Interquartile range = 12.5–21.4, *n* = 185). We also tried to position the pipette at a similar distance to each arteriole. The median distance was 38.0 μm (Interquartile range = 29.9–50.3, *n* = 185).

We first tested the effects of several different transporter substrates, including L-Glu (100 μM or 1 mM), D-Asp (100 μM, or 1 mM), and L-THA (1 mM). These compounds are substrates for the Na^+^-dependent glutamate transporters, but they also activate glutamate receptors (Arriza et al., 1994; Erreger et al., 2007). After all experiments were complete, we used an automated image analysis method to analyze these data (Methods: Image Analysis). In brief, the program drew up to 8 lines across the blood vessel, filtered images not in the plane/focus, calculated the average diameter from the 3 s of baseline data obtained prior to pressure application, and then generated kymographs of changes in blood vessel diameter over time for each of the lines. These kymographs were converted into two-dimensional graphs of arteriole diameter (normalized to baseline) as a function of time.

The analyses of the effects of L-Glu (100 μM or 1 mM), D-Asp (100 μM, or 1 mM), and L-THA (1 mM) are from relatively few animals and were done as pilot experiments. Therefore, the data were explored visually with no formal statistical inference. These data are included to explain the development of the methods. The boxplots suggest some trends toward larger peaks (AUC) after application of Glu, D-Asp, or 1 mM L-THA compared to saline (Figure 1). Similarly, there are some trends for larger effects on the maximum change observed after application of these substrates, however, as the analyses were likely underpowered to detect relevant biological effects, no statistical comparisons were made. Based on these observations, we focused our subsequent analyses on the effects of lower concentrations of L-THA (100 μM). At this concentration, L-THA does not activate ionotropic glutamate receptors (Erreger et al., 2007).

**FIGURE 4:**
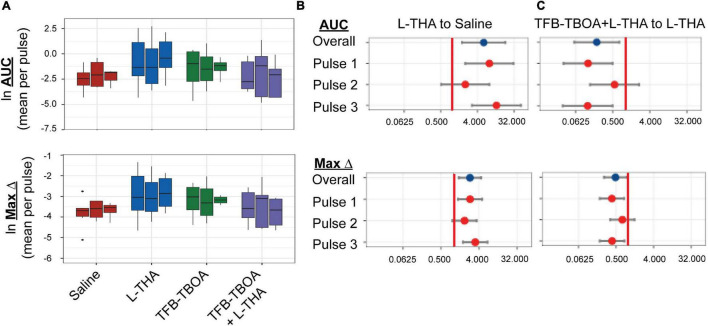
Effects of saline, L-THA, TFB-TBOA, and TFB-TBOA with L-THA on arteriole diameter. Saline, L-THA (100 μM), TFB-TBOA (2 μM), or a combination of TFB-TBOA with L-THA were picospritzed near cortical arterioles. **(A)** Natural log (ln)-transformed raw data are presented as boxplots, using the mean of repeated measures from each animal. Raw values are from dilations (positive values), however, as data are ln-transformed, values > 1 are positive while values between 0 and 1 will appear on this scale as negative values **(B,C)** forest plots are included to visualize the results of the model and show the mean differences expressed as ratios of the effect observed with L-THA over that observed with saline **(B)** or TFB-TBOA with L-THA over that observed with L-THA **(C)**. The redline is at 1 and represents no difference. The data for saline are from 6 animals (3 males, 3 females) and are the same data as that presented in Figures 1, 5–9, the data for L-THA are from 12 animals (7 males, 5 females) and are also the same as those presented in Figures 5–9. The data for TFB-TBOA are from 4 animals (2 males, 2 females) and the data for TFB-TBOA with L-THA are from 5 animals (3 males, 2 females).

**FIGURE 5:**
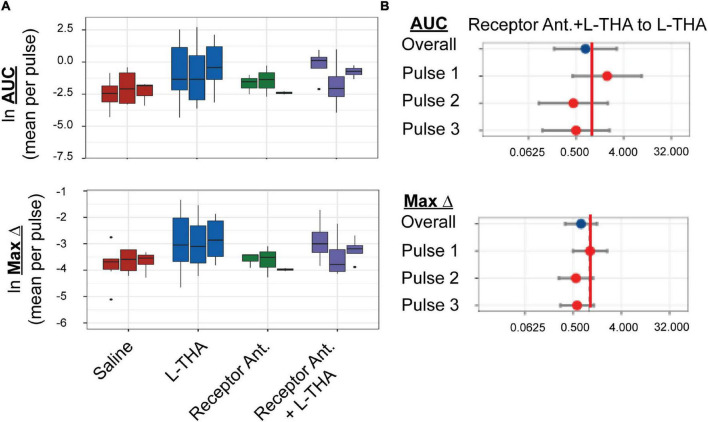
Effects of saline, L-THA, receptor antagonists, and receptor antagonists with L-THA on arteriole diameter. Saline, L-THA (100 μM), receptor antagonist cocktail [DNQX (10 μM)/D-APV (50 μM)/MCPG (1 mM)/R-Baclofen (100 μM)], or a combination of receptor antagonist cocktail with L-THA were picospritzed near cortical arterioles. **(A)** Data are presented as boxplots. **(B)** Forest plots are also included to show the mean differences expressed as ratios of the effect of Receptor Antagonists with L-THA over that observed with L-THA. The redline is at 1 and represents no difference. The data for saline and L-THA are described in Figure 4 legend, the data for receptor antagonists are from 3 animals (1 male, 2 females) and the data for receptor antagonists with L-THA are from 4 animals (1 male, 3 females).

**FIGURE 6:**
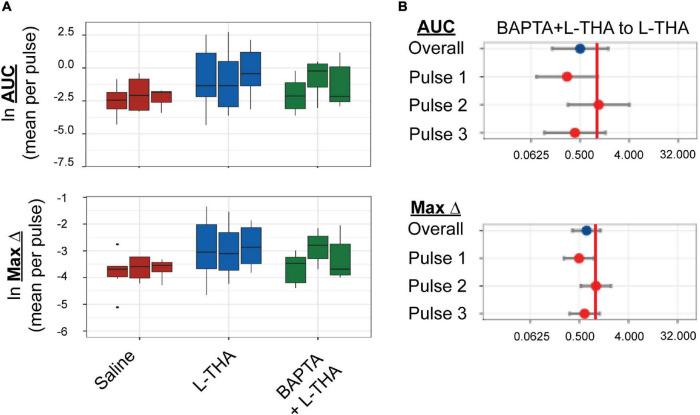
Effects of saline, L-THA, or L-THA with BAPTA pretreatment on arteriole diameter. Saline, L-THA (100 μM), or L-THA after pre-treatment with BAPTA (see methods) were picospritzed near cortical arterioles. **(A)** Data are presented as boxplots. **(B)** Forest plots are also included to show the mean differences expressed as ratios of the effect of BAPTA with L-THA over that observed with L-THA. The redline is at 1 and represents no difference. The data for saline and L-THA are described in Figure 4 legend, the data for BAPTA with L-THA are from 6 animals (2 males, 4 females).

**FIGURE 7:**
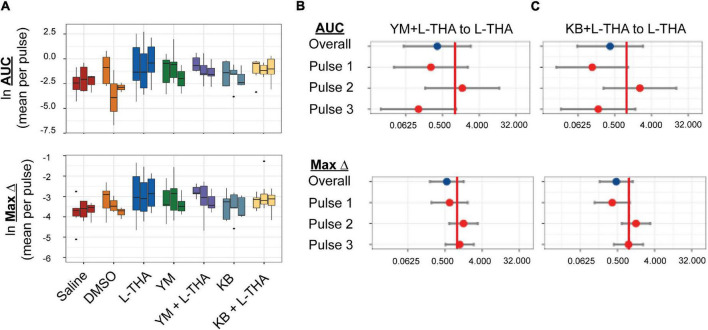
Effects of saline, L-THA, YM-244769, KB-R7943, and YM or KB with L-THA on arteriole diameter. Saline, L-THA (100 μM), YM-244769 (1 μM), KB-R7943 (15 μM), or a combination of YM or KB with L-THA were picospritzed near cortical arterioles. **(A)** Data are presented as boxplots. Forest plots are also included to show the mean differences expressed as ratios of the effect observed with YM with L-THA **(B)** or KB with L-THA **(C)** over that observed with L-THA. The redline is at 1 and represents no difference. The data for saline and L-THA are described in Figure 4 legend, the data for YM are from 6 animals (3 males, 3 females), the data for YM with L-THA are from 6 animals (2 males, 4 females), the data from KB are from 5 animals (1 male, 4 females) and the data for KB with L-THA are from 7 animals (5 males, 2 females).

**FIGURE 8:**
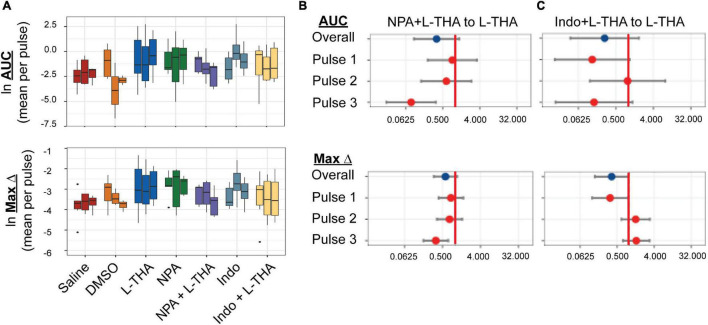
Effects of saline, L-THA, L-NPA, Indomethacin, and L-NPA or Indomethacin with L-THA on arteriole diameter. Saline, L-THA (100 μM), L-NPA (2 μM), Indomethacin (10 μM), or a combination of NPA or Indomethacin with L-THA were picospritzed near cortical arterioles. **(A)** Data are presented as boxplots. Forest plots are also included to show the mean differences expressed as ratios of the effect observed with NPA with L-THA **(B)** or Indomethacin with L-THA **(C)** over that observed with L-THA. The redline is at 1 and represents no difference. The data for saline and L-THA are described in Figure 4 legend, the data for NPA are from 5 animals (4 males, 1 female), the data for NPA with L-THA are from 5 animals (3 males, 2 females), the data from Indomethacin are from 8 animals (3 males, 5 females) and the data for Indomethacin with L-THA are from 6 animals (3 males, 3 females).

To test the hypothesis that L-THA causes an increase in arteriole diameter in excess of that observed after application of saline, we identified the largest dilation, as defined by the peak with the largest area (AUC), and measured the maximum change in diameter, time to the maximum change in diameter, and the duration of this peak. We report changes in arteriole diameter both in terms of max change and largest AUC as the area provides an integration of arteriole dilation over time whereas the max change allows one to directly calculate the size of the effect on arteriole diameter as these provide somewhat different information. The experiments with 100 μM L-THA and vehicle were inter-mixed with studies of the effects of various pharmacologic inhibitors. Across Figures 4, 5–8, the saline and L-THA groups represent identical animals and are repeated for purposes of comparison. The boxplots (Figure 4A) suggest a substantial response to L-THA at each pulse, both for AUC and Max Change. As indicated in the methods, the overall statistical model provided evidence of different effects for at least some of the pharmacologic agents by pulse. Therefore, we have compared the data for each treatment at each individual pulse. Tables 1–4 and the forest plots in Figures 4, 5–8 thus show the overall effect of each agent, summarized across the pulses, as well as the effect for each pulse. The forest plot (Figure 4B) present the model-based estimates of the mean difference, after exponentiating, to give a ratio of the effect of L-THA over that observed for saline. The top row shows the combination of the three pulses with individual pulses in the lower rows. The error bars show 95% CIs. Note that when a 95% CI for the ratio includes a value of 1.0, the null hypothesis of no effect cannot be ruled out at the 0.05 level, i.e., *p* > 0.05. When the effects of the three pulses are combined, the mean area of the largest dilation, area under the curve (AUC), caused by L-THA was 5.7-fold larger than that observed after saline (95% CI, 1.7–19, *p* = 0.006, Figure 4B and Table 1). Similarly, the maximum change in arteriole diameter was 2.4-fold larger than that observed with saline (95% CI, 1.3–4.5, *p* = 0.005, Figure 4B and Table 3). The mean L-THA-induced increase in blood vessel diameter was 5.8%. As blood flow should correlate to the fourth power of radius (Poiseuille’s equation) (Petzold et al., 2008), this change in arteriole diameter represents an ∼25% increase in blood flow and is similar to that elicited by sensory stimulation (whisker stimulation or odorants) (Petzold et al., 2008; Masamoto et al., 2015; Tran and Gordon, 2015). The median time to peak observed after 100 μM L-THA was 44.60 s (interquartile range = 26.0–67.5, *n* = 283) and the median duration of these peaks was 18.2 s (interquartile range = 6.1–41.1, *n* = 283). These data show that pulse application of L-THA causes a substantial increase in arteriole diameter and that this effect cannot be attributed the mechanical force of pressure application. Although we tested the effects of two different concentrations of L-Glu, D-Asp, and L-THA, it is not possible to test for concentration-dependence of these effects with so few concentrations.

**TABLE 1:** Combined pulses – Area under the curve^1^.

Contrast	Difference (natural log scale)			Ratio		
	Mean^2^	SE^3^	*P*-value^4^	Estimate^5^	95% CI^6^	
					Lower	Upper
DMSO to Saline	0.636	0.745	0.396	1.889	0.429	8.320
L-THA to Saline	1.741	0.617	0.006	5.706	1.670	19.490
TFB-TBOA to Saline	0.998	0.771	0.200	2.713	0.583	12.616
TFB-TBOA + L-THA to Saline	0.151	0.730	0.836	1.163	0.272	4.979
TFB-TBOA + L-THA to L-THA	–1.590	0.633	0.014	0.204	0.058	0.721
Receptor Ant. to Saline	0.251	0.857	0.771	1.285	0.233	7.090
Receptor Ant. + L-THA to Saline	1.456	0.775	0.064	4.288	0.916	20.080
Receptor Ant. + L-THA to L-THA	–0.286	0.685	0.678	0.751	0.192	2.940
BAPTA to Saline	1.054	0.696	0.134	2.868	0.716	11.480
BAPTA to L-THA	–0.688	0.594	0.251	0.503	0.154	1.640
YM to DMSO	0.446	0.731	0.544	1.562	0.364	6.690
YM + L-THA to DMSO	0.750	0.730	0.308	2.116	0.494	9.060
YM + L-THA-D to L-THA-Saline	–0.992	0.956	0.303	0.371	0.055	2.490
KB to DMSO	0.049	0.756	0.949	1.050	0.233	4.740
KB + L-THA to DMSO	0.781	0.705	0.272	2.184	0.536	8.890
KB + L-THA-D to L-THA-Saline	–0.961	0.937	0.309	0.383	0.059	2.470
NPA to Saline	1.367	0.734	0.066	3.923	0.909	16.930
NPA + L-THA to Saline	0.699	0.733	0.343	2.012	0.467	8.670
NPA + L-THA to L-THA	–1.042	0.637	0.106	0.353	0.099	1.250
Indo to DMSO	1.038	0.686	0.134	2.824	0.720	11.080
Indo + L-THA to DMSO	0.422	0.726	0.563	1.525	0.359	6.480
Indo + L-THA-D to L-THA-Saline	–1.320	0.953	0.170	0.267	0.040	1.780

*^1^Results based on a mixed effects model using the natural log of AUC as the outcome and including all experimental conditions; ^2^Difference of means (natural log scale), equivalently ln(Ratio); ^3^Standard error; ^4^P-value for Wald test; ^5^Exponentiated Difference of Means; ^6^95% confidence interval.*

**TABLE 2:** Individual pulses – Area under the curve^1^.

Contrast	Pulse	Difference (natural log scale)			Ratio		
		Mean^2^	SE^3^	*P*-value^4^	Estimate^5^	95% CI^6^	
						Lower	Upper
DMSO to Saline	1	1.632	0.809	0.046	5.116	1.030	25.400
	2	–0.382	0.831	0.647	0.682	0.132	3.540
	3	0.658	0.858	0.445	1.931	0.354	10.550
L-THA to Saline	1	2.062	0.691	0.003	7.860	2.003	30.836
	2	0.698	0.691	0.314	2.009	0.512	7.885
	3	2.465	0.702	0.001	11.763	2.934	47.154
TFB-TBOA to Saline	1	1.401	0.844	0.100	4.059	0.761	21.650
	2	0.316	0.846	0.710	1.371	0.256	7.335
	3	1.278	0.852	0.137	3.588	0.662	19.438
TFB-TBOA + L-THA to Saline	1	–0.016	0.803	0.984	0.984	0.200	4.833
	2	0.106	0.805	0.895	1.112	0.226	5.484
	3	0.364	0.815	0.656	1.440	0.286	7.238
TFB-TBOA + L-THA to L-THA	1	–2.078	0.693	0.003	0.125	0.032	0.495
	2	–0.591	0.693	0.395	0.554	0.140	2.188
	3	–2.101	0.705	0.004	0.122	0.030	0.494
Receptor Ant. to Saline	1	1.123	0.933	0.231	3.073	0.484	19.510
	2	0.392	0.935	0.676	1.480	0.232	9.440
	3	–0.762	0.995	0.445	0.467	0.065	3.340
Receptor Ant. + L-THA to Saline	1	2.722	0.862	0.002	15.209	2.759	83.830
	2	–0.118	0.862	0.892	0.889	0.161	4.910
	3	1.763	0.844	0.039	5.831	1.093	31.100
Receptor Ant. + L-THA to L-THA	1	0.660	0.760	0.387	1.935	0.429	8.720
	2	–0.816	0.759	0.285	0.442	0.098	1.990
	3	–0.702	0.738	0.344	0.496	0.115	2.140
BAPTA to Saline	1	0.816	0.768	0.290	2.262	0.494	10.360
	2	0.793	0.774	0.308	2.210	0.477	10.240
	3	1.552	0.771	0.047	4.720	1.024	21.760
BAPTA to L-THA	1	–1.245	0.651	0.059	0.288	0.079	1.050
	2	0.095	0.657	0.885	1.100	0.299	4.040
	3	–0.913	0.653	0.165	0.401	0.110	1.470
YM to DMSO	1	0.064	0.797	0.937	1.066	0.220	5.170
	2	1.348	0.803	0.096	3.848	0.783	18.910
	3	–0.074	0.825	0.929	0.929	0.181	4.760
YM + L-THA to DMSO	1	0.711	0.799	0.376	2.035	0.417	9.930
	2	1.126	0.803	0.164	3.083	0.628	15.140
	3	0.412	0.821	0.617	1.510	0.297	7.670
YM + L-THA-D to L-THA-Saline	1	–1.351	1.057	0.203	0.259	0.032	2.100
	2	0.428	1.059	0.687	1.534	0.188	12.510
	3	–2.053	1.080	0.060	0.128	0.015	1.090
KB to DMSO	1	–0.065	0.804	0.936	0.937	0.190	4.630
	2	0.454	0.828	0.584	1.575	0.305	8.140
	3	–0.243	0.849	0.775	0.784	0.146	4.210
KB + L-THA to DMSO	1	0.086	0.753	0.909	1.090	0.244	4.860
	2	1.426	0.778	0.070	4.162	0.890	19.470
	3	0.831	0.802	0.302	2.295	0.469	11.220
KB + L-THA-D to L-THA-Saline	1	–1.976	1.022	0.056	0.139	0.018	1.050
	2	0.728	1.041	0.486	2.071	0.264	16.270
	3	–1.634	1.066	0.128	0.195	0.024	1.610
NPA to Saline	1	1.741	0.811	0.034	5.704	1.145	28.427
	2	0.754	0.819	0.359	2.125	0.419	10.762
	3	1.606	0.820	0.053	4.981	0.982	25.273
NPA + L-THA to Saline	1	1.904	0.805	0.020	6.714	1.362	33.091
	2	0.201	0.822	0.807	1.223	0.240	6.227
	3	–0.007	0.811	0.993	0.993	0.199	4.949
NPA + L-THA to L-THA	1	–0.158	0.695	0.821	0.854	0.215	3.389
	2	–0.497	0.713	0.487	0.608	0.148	2.497
	3	–2.472	0.699	0.001	0.084	0.021	0.338
Indo to DMSO	1	–0.216	0.733	0.769	0.806	0.188	3.450
	2	2.262	0.757	0.003	9.600	2.141	43.050
	3	1.068	0.779	0.173	2.910	0.623	13.600
Indo + L-THA to DMSO	1	0.054	0.785	0.945	1.056	0.222	5.010
	2	0.559	0.819	0.496	1.749	0.346	8.850
	3	0.652	0.796	0.414	1.919	0.396	9.300
Indo + L-THA-D to L-THA-Saline	1	–2.007	1.046	0.057	0.134	0.017	1.070
	2	–0.046	1.054	0.965	0.955	0.118	7.710
	3	–1.906	1.078	0.080	0.149	0.018	1.260

*^1^Results based on a mixed effects model using the natural log of AUC as the outcome and including all experimental conditions; ^2^Difference of means (natural log scale), equivalently ln(Ratio); ^3^Standard error; ^4^P-value for Wald test; ^5^Exponentiated Difference of Means; ^6^95% confidence interval.*

**TABLE 3:** Combined pulses – Max change^1^.

Contrast	Difference (natural log scale)			Ratio		
	Mean^2^	SE^3^	*P*-value^4^	Estimate^5^	95% CI^6^	
					Lower	Upper
DMSO to Saline	0.450	0.369	0.226	1.568	0.753	3.268
L-THA to Saline	0.892	0.306	0.005	2.439	1.328	4.481
TFB-TBOA to Saline	0.593	0.385	0.127	1.810	0.841	3.895
TFB-TBOA + L-THA to Saline	0.164	0.363	0.653	1.178	0.571	2.428
TFB-TBOA + L-THA to L-THA	–0.728	0.316	0.024	0.483	0.257	0.907
Receptor Ant. to Saline	–0.085	0.426	0.842	0.918	0.393	2.140
Receptor Ant. + L-THA to Saline	0.538	0.386	0.167	1.713	0.794	3.690
Receptor Ant. + L-THA v L-THA	–0.353	0.342	0.305	0.702	0.355	1.390
BAPTA to Saline	0.499	0.347	0.154	1.647	0.826	3.280
BAPTA to L-THA	–0.393	0.297	0.190	0.675	0.374	1.220
YM to DMSO	0.154	0.363	0.674	1.166	0.565	2.400
YM + L-THA to DMSO	0.301	0.363	0.409	1.352	0.656	2.790
YM + L-THA-D to L-THA-Saline	–0.590	0.475	0.217	0.554	0.215	1.430
KB to DMSO	–0.185	0.377	0.625	0.831	0.392	1.760
KB + L-THA to DMSO	0.130	0.351	0.711	1.139	0.566	2.290
KB + L-THA-D to L-THA-Saline	–0.761	0.465	0.106	0.467	0.185	1.180
NPA to Saline	0.893	0.365	0.017	2.442	1.181	5.050
NPA + L-THA to Saline	0.359	0.364	0.328	1.432	0.693	2.960
NPA + L-THA to L-THA	–0.533	0.318	0.098	0.587	0.312	1.110
Indo to DMSO	0.234	0.341	0.496	1.263	0.640	2.494
Indo + L-THA to DMSO	–0.111	0.362	0.759	0.895	0.435	1.839
Indo + L-THA-D to L-THA-Saline	–1.003	0.474	0.037	0.367	0.143	0.942

*^1^Results based on a mixed effects model using the natural log of Max change as the outcome and including all experimental conditions; ^2^Difference of means (natural log scale), equivalently ln(Ratio); ^3^Standard error; ^4^P-value for Wald test; ^5^Exponentiated Difference of Means; ^6^95% confidence interval.*

**TABLE 4:** Individual pulses – Max change^1^.

Contrast	Pulse	Difference (natural log scale)			Ratio		
		Mean^2^	SE^3^	*P*-value^4^	Estimate^5^	95% CI^6^	
						Lower	Upper
DMSO to Saline	1	0.647	0.392	0.102	1.909	0.878	4.150
	2	0.346	0.402	0.391	1.413	0.637	3.130
	3	0.357	0.412	0.388	1.430	0.632	3.230
L-THA to Saline	1	0.899	0.332	0.008	2.457	1.274	4.740
	2	0.594	0.335	0.079	1.810	0.933	3.514
	3	1.182	0.338	0.001	3.261	1.669	6.372
TFB-TBOA to Saline	1	0.664	0.411	0.109	1.944	0.860	4.391
	2	0.344	0.414	0.408	1.410	0.621	3.204
	3	0.772	0.416	0.066	2.164	0.949	4.935
TFB-TBOA + L-THA to Saline	1	–0.024	0.389	0.950	0.976	0.451	2.113
	2	0.247	0.393	0.530	1.280	0.588	2.790
	3	0.268	0.396	0.499	1.308	0.597	2.867
TFB-TBOA + L-THA to L-THA	1	–0.923	0.339	0.008	0.397	0.203	0.778
	2	–0.346	0.339	0.309	0.707	0.361	1.386
	3	–0.914	0.344	0.009	0.401	0.203	0.793
Receptor Ant. to Saline	1	0.209	0.453	0.645	1.233	0.502	3.030
	2	0.012	0.456	0.980	1.012	0.409	2.500
	3	–0.477	0.479	0.321	0.620	0.240	1.600
Receptor Ant. + L-THA to Saline	1	0.937	0.417	0.027	2.553	1.116	5.840
	2	0.019	0.420	0.964	1.019	0.443	2.340
	3	0.658	0.412	0.114	1.931	0.852	4.380
Receptor Ant. + L-THA to L-THA	1	0.038	0.371	0.918	1.039	0.498	2.170
	2	–0.575	0.370	0.124	0.563	0.270	1.170
	3	–0.524	0.362	0.152	0.592	0.288	1.220
BAPTA to Saline	1	0.187	0.372	0.616	1.206	0.576	2.521
	2	0.601	0.377	0.114	1.825	0.864	3.853
	3	0.708	0.375	0.062	2.030	0.965	4.271
BAPTA to L-THA	1	–0.712	0.318	0.028	0.491	0.261	0.923
	2	0.008	0.321	0.980	1.008	0.534	1.904
	3	–0.474	0.319	0.141	0.623	0.330	1.173
YM to DMSO	1	0.104	0.389	0.790	1.109	0.513	2.400
	2	0.227	0.391	0.563	1.254	0.578	2.720
	3	0.131	0.399	0.744	1.140	0.517	2.510
YM + L-THA to DMSO	1	0.465	0.390	0.235	1.593	0.735	3.450
	2	0.212	0.390	0.589	1.236	0.570	2.680
	3	0.227	0.398	0.570	1.255	0.570	2.760
YM + L-THA-D to L-THA-Saline	1	–0.434	0.512	0.399	0.648	0.235	1.790
	2	–0.382	0.514	0.459	0.682	0.246	1.890
	3	–0.955	0.522	0.070	0.385	0.137	1.080
KB to DMSO	1	–0.233	0.395	0.557	0.792	0.361	1.737
	2	–0.248	0.404	0.541	0.780	0.350	1.739
	3	–0.074	0.412	0.857	0.928	0.410	2.101
KB + L-THA to DMSO	1	–0.088	0.369	0.813	0.916	0.440	1.907
	2	0.182	0.378	0.632	1.199	0.566	2.539
	3	0.297	0.388	0.445	1.346	0.625	2.901
KB + L-THA-D to L-THA-Saline	1	–0.987	0.496	0.049	0.373	0.139	0.998
	2	–0.412	0.505	0.417	0.662	0.243	1.803
	3	–0.885	0.514	0.088	0.413	0.149	1.144
NPA to Saline	1	0.905	0.392	0.023	2.472	1.135	5.381
	2	0.786	0.398	0.051	2.195	0.997	4.832
	3	0.987	0.398	0.015	2.683	1.219	5.903
NPA + L-THA to Saline	1	0.668	0.390	0.090	1.950	0.900	4.228
	2	0.289	0.399	0.471	1.335	0.605	2.945
	3	0.120	0.394	0.761	1.128	0.516	2.465
NPA + L-THA to L-THA	1	–0.231	0.340	0.498	0.794	0.405	1.557
	2	–0.305	0.347	0.381	0.737	0.371	1.466
	3	–1.062	0.341	0.002	0.346	0.176	0.681
Indo to DMSO	1	–0.136	0.359	0.707	0.873	0.428	1.783
	2	0.456	0.368	0.218	1.578	0.761	3.273
	3	0.381	0.377	0.314	1.463	0.694	3.086
Indo + L-THA to DMSO	1	–0.193	0.384	0.617	0.825	0.385	1.769
	2	–0.227	0.388	0.560	0.797	0.370	1.720
	3	0.085	0.397	0.830	1.089	0.496	2.391
Indo + L-THA-D to L-THA-Saline	1	–1.092	0.508	0.034	0.336	0.123	0.918
	2	–0.820	0.512	0.112	0.440	0.160	1.216
	3	–1.097	0.521	0.038	0.334	0.119	0.938

*^1^Results based on a mixed effects model using the natural log of Max change as the outcome and including all experimental conditions; ^2^Difference of means (natural log scale), equivalently ln(Ratio); ^3^Standard error; ^4^P-value for Wald test; ^5^Exponentiated Difference of Means; ^6^95% confidence interval.*

**FIGURE 9:**
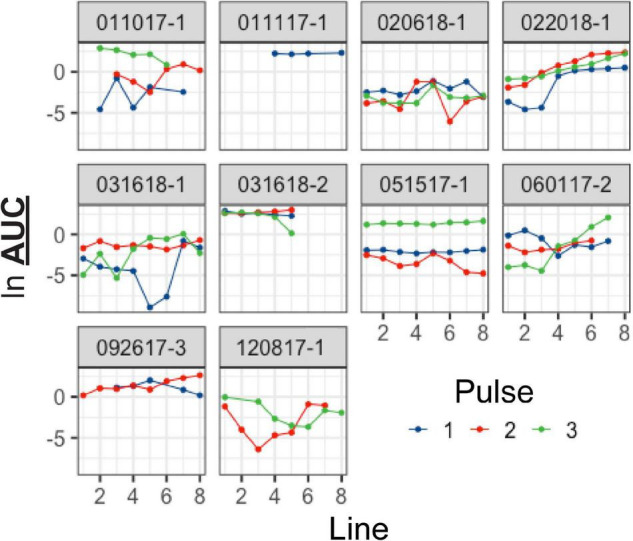
Visual exploration of responders and non-responders. Arteriole responses (ln-transformed AUC) are plotted for each individual arteriole in response to 100 μM L-THA. The responses at each individual arteriole segment (lines) are represented as points. Each graph represents data obtained from an individual animal and the effects of different pulses are shown in different colors. Note two animals are not presented. On the Ln scale, the mean response is –0.55 (95% CI –1.25 to 0.15).

As we observed considerable variability in responses to L-THA (and other agents) both within and between animals, we examined several possible sources that might modulate these results. First, we tested for overall sex differences in the responsiveness of arterioles to L-THA. Since the study was not designed to test hypotheses regarding different responses to L-THA for males and females, we refit the model used in the main analysis separately for the two biological sexes. For saline, the study included 3 females and 3 males; for L-THA, there were 5 females and 7 males, and for L-THA+ TFB-TBOA there were 2 females and 3 males. Averaged across pulses, the mean AUC after THA was 5.8-fold larger than in saline in females (95% CI 0.95–35.5, *p* = 0.056) and 12.9-fold larger in males (95% CI 1.7–97.6, *p* = 0.015). The maximum increase in diameter observed after L-THA was 2.25-fold larger than in saline for females (95% CI 0.93–5.45, *p* = 0.071), and 3.20 fold larger in males (95% CI 1.20–8.52, *p* = 0.021). Thus, the response to L-THA was substantial for both sexes. It is also possible that there is uncontrolled anatomic variability. The spread of L-THA is likely to be restricted due to the focal application and by the robust clearance mediated by Na^+^-dependent glutamate transporters (Garthwaite, 1985; Herman and Jahr, 2007). Figure 9 shows the L-THA-induced changes in diameter that were observed at individual locations (8) along the same arteriole. We depict these responses across multiple animals and with each individual L-THA application (pulse, typically 3). This illustrates three sources of variability, between-animal, between pulses within animals, and across lines within animals. For example, animals examined on 031618-2 and 092617-3 showed a substantially stronger response than, for example, 020618-1. Formal statistical methods for discerning latent classes of responders and non-responders to L-THA would require substantially larger sample sizes than used here. As indicated above, the statistical model detected variability in the response by pulse, noted previously in the boxplots and evident here as well in some animals, e.g., 051517-1. Moreover, there was considerable variability in the functional form of the response across the lines. For example, 031618-2 and 051517-1 shows little variation across lines for the individual pulses while 022018-1 tends to increase with higher values for line 6–8 compared to 1–3.

We next tested whether the L-THA-evoked increases in vessel diameter could be blocked by inhibiting glutamate transporters. We focally applied (2S,3S)-3-[3-[4-(trifluoromethyl)-benzoylamino]benzyloxy]-aspartate (TFB-TBOA) and TFB-TBOA in combination with L-THA (Figure 4) at descending arterioles. TFB-TBOA is a pan-inhibitor of the Glu transporters with IC_50_ values of ∼20 nM for the glial Glu transporters, 300 nM for EAAC1, and no interactions with Glu receptors at concentrations up to 100 μM (Shimamoto et al., 2004). Expressed as a ratio compared to saline, the dilations observed after application of TFB-TBOA trended toward increases in AUC (2.7-fold 95%, CI 0.6–12.6, *p* = 0.20, Table 1) and in Max Change (1.8-fold 95% CI 0.8–3.9, *p* = 0.12) (see Figure 4B, Table 3). When combined with L-THA, levels were near those of saline, i.e., for AUC (1.2-fold, 95% CI 0.3–4.9, *p* = 0.84) and Max Change (1.2-fold, 95% CI 0.6–2.4, *p* = 0.65) (Tables 1, 3). Compared to L-THA alone, the effects observed after TFB-TBOA with L-THA tended to be lower for AUC (0.20-fold, 95% CI 0.06-0.72, p = 0.014) and Max Change (0.48-fold, 95% CI 0.26–0.91, *p* = 0.024) (see forest plots, Figure 4C). These results indicate that the effects of L-THA are blocked by TFB-TBOA. We also examined possible sex differences in this group. Averaged across pulses, the mean AUC after L-THA with TFB-TBOA was 0.27-fold that of L-THA in females (95% CI 0.04–1.87, *p* = 0.18) and 0.13-fold that of L-THA in males (95% CI 0.02–0.98, *p* = 0.048). Averaged across pulses, the maximum change after L-THA with TFB-TBOA was 0.53-fold of L-THA alone in females (95% CI 0.20–1.39, *p* = 0.19) and 0.41-fold of L-THA in males (95% CI 0.15–1.07, *p* = 0.068). Thus, within the precision of our study design, the data suggest TFB-TBOA substantially blocks the effects of L-THA in both sexes.

We tested the effects of a cocktail of iGluR antagonists, including DNQX (10 μM), D-APV (50 μM), MCPG (1 mM), and baclofen (100 μM). An identical strategy (and concentrations) was employed by Petzold et al. to demonstrate a TBOA and dihydrokainate-sensitive (i.e., glutamate transport-dependent) component to olfaction-evoked neurovascular coupling (Petzold et al., 2008). These receptor antagonists were also dissolved in saline. Although the sample sizes are small and the confidence intervals wide, this combination of receptor antagonists appeared to have minimal effect on arteriole diameter (AUC or Max Change, Figure 5) when expressed as a ratio of that observed with saline (1.3 or 0.9, respectively, Tables 1, 3). When applied in combination with L-THA, peaks were nearly as big as those observed after THA alone (Area 4.3 versus 5.7, and max change 1.7 versus 2.4). As can been seen in the forest plots (Figure 5B), this cocktail of receptor antagonists trended toward a small decrease.

To determine if the effects of L-THA were dependent upon increases in astrocytic Ca^2+^, we tested if the effects of L-THA could be blocked by application of a Ca^2+^ chelator. As others have done, we relied on the observation that cortical surface loading of various acetyl-methyl ester indicators (e.g., Oregon-green BAPTA; OGB) and chelators (e.g., BAPTA-AM) favor distribution of dye throughout the astrocytic syncytium (Nimmerjahn et al., 2004; Navarrete et al., 2012). We applied BAPTA-AM (20 μM) to the cortical surface for 20 min prior to sealing the cranial window and moving the animal to the microscope. We did not observe overall group differences between the effects of L-THA alone and L-THA after application of BAPTA, but there is a trend toward a decrease after the first pulse (see Figure 6). It is conceivable that BAPTA is cleared after longer times.

Na^+^-dependent glutamate uptake causes a rapid increase in intracellular Na^+^ and astrocytic depolarization (Bergles and Jahr, 1997; Langer et al., 2017). The Na^+^/Ca^2+^ exchangers (NCX) exchange 3 Na^+^ ions for 1 Ca^2+^ ion (Minelli et al., 2007; Pappalardo et al., 2014) in either direction depending on membrane potential and the ionic gradients of Na^+^ and Ca^2+^. The reversal potential of the NCX isoforms is close to the astrocytic resting membrane potential (Kirischuk et al., 1997; Reyes et al., 2012), thus the exchangers frequently operate in the so-called “reverse-mode” (Ca^2+^ in /Na^+^ out) (Blaustein et al., 2002). These transporters have turnover numbers of 2000-5000/sec giving them almost channel-like kinetic properties (Blaustein and Lederer, 1999). Na^+^-dependent Glu uptake couples to increases in intracellular Ca^2+^ through this reversed operation in cell lines (Magi et al., 2013), cultured astrocytes (Reyes and Parpura, 2008; Rojas et al., 2013; Parpura et al., 2016), and in processes in organotypic cultures of rat hippocampus (Jackson and Robinson, 2015). Here, we tested the effects of two structurally dissimilar inhibitors of the reversed-mode of these exchangers, YM244769 and KB-R7943, on arteriole diameter alone and the presence of L-THA. These drugs were dissolved in DMSO and then saline to final concentration of 0.1% DMSO. Unexpectedly, compared to Saline, DMSO may have caused a small increase in AUC (Ratio of 1.89, 95% CI 0.43–8.3) and Max Change (Ratio of 1.57, 95% CI 0.75–3.27) (Tables 1, 3), and we have no direct information about effects of L-THA alone in the presence of DMSO relative to saline. The effects of YM244769 and KB-R7943 alone are reported relative to saline with DMSO and then normalized to saline alone. The effects of YM244769 and KB-R7943 in combination with L-THA are reported relative to Saline with DMSO, and then normalized to the effect of L-THA alone compared to Saline Figure 7, see Tables 1–4). Our results are inconclusive, however, the findings, particularly for pulses 1 and 3, suggest that this mechanism should not be ruled out.

Several groups have demonstrated that pharmacological inhibition or genetic deletion of neuronal nitric oxide synthetase (nNOS) blocks the neurovascular response (Faraci and Breese, 1993; Yang et al., 2003; Kitaura et al., 2007; Toda et al., 2009). Although the levels of nNOS are higher in subsets of neurons, both mRNA and protein for the various nNOS isoforms are found in astrocytes (Kugler and Drenckhahn, 1996; Togashi et al., 1997; Zhang et al., 2014; Munoz et al., 2015). Based on these observations, we tested the effects of the selective inhibitor of nNOS inhibitor, Nω-propyl-L-arginine (L-NPA) (2 μM), which was dissolved in saline (Zhang et al., 1997). The effects of NPA alone or in combination with L-THA are presented in Figure 8 (Tables 1–4 for summary data). Compared to saline, the peaks observed after NPA trended toward being larger (AUC 3.9-fold, 95% CI 0.91–16.9, *p* = 0.07, Maximum change 2.4-fold, 95% CI 1.2–5.0, *p* = 0.02). When combined with L-THA, the effects were similar to those of Saline, i.e., for AUC (2.0-fold, 95% CI 0.5–8.7, *p* = 0.33) and for Max Change (1.4-fold 95% CI 0.7–3.0, *p* = 0.33; see forest plots, Figure 8B).

The mRNA and protein for the enzymes that generate archidonic acid (phospholipase A2, PLA2) and the metabolite PGE2 (cyclooxygenase, Cox1,2) have been identified in astrocytes (Takano et al., 2006; Gordon et al., 2008; Shi et al., 2008; Zhang et al., 2014). Inhibition of these enzymes blocks the vasodilation induced by uncaging Ca^2+^ in astrocytes and/or attenuates stimulus-evoked increases in blood flow (Takano et al., 2006; Gordon et al., 2008; Shi et al., 2008). To determine if prostaglandin E2 (PGE_2_) is involved in the effects of L-THA, we tested the effects of inhibition of both cytochrome oxidase 1 and 2 (Cox1 & 2) with the pan-Cox inhibitor indomethacin (10 μM), which was also dissolved in DMSO and then saline (Mulligan and MacVicar, 2004). While the sizes of peaks observed after application of indomethacin trend toward being smaller than those observed after L-THA alone (Figure 8C and Tables 1–4), we did not examine the effects of L-THA in the presence of 0.1% DMSO.

## Discussion

The regulation of neurovascular coupling is complex, with multiple cell types participating depending on context and localization, including excitatory and inhibitory neurons, pericytes, astrocytes, vascular smooth muscle cells, and endothelia (Iadecola and Nedergaard, 2007; Koehler et al., 2009; Attwell et al., 2010; Hamilton et al., 2010; Petzold and Murthy, 2011; Filosa and Iddings, 2013; Gurden, 2013; Stobart and Anderson, 2013; Hillman, 2014; Howarth, 2014; Filosa et al., 2015; Hill et al., 2015; Tran and Gordon, 2015; Mishra et al., 2016; Uhlirova et al., 2016; Mateo et al., 2017). Moreover, there is evidence of cross-talk between these cells and the various signals that modulate the responses.

Several studies have implicated a role for glutamate transporters in the neurovascular response. Genetic or pharmacologic inhibition of glutamate transport reduces neural activity-induced increases in blood flow or energy delivery. For example, antisense knockdown of GLAST reduces whisker stimulation-induced 2-deoxyglucose accumulation in barrel cortex of rats (Cholet et al., 2001), and genetic deletion of either GLT-1 or GLAST reduces whisker-stimulation-induced accumulation of 2-deoxyglucose in young (10 days of age) mice (Voutsinos-Porche et al., 2003). A similar effect of GLT-1 knockout was observed with visual stimulation in the superior colliculus (Herard et al., 2005). Odor-induced increases in intrinsic optical signals in rats (Gurden et al., 2006) or blood flow in mice (Petzold et al., 2008) are attenuated by pharmacologically blocking glutamate uptake with DL-*threo*-ß-benzyloxyaspartic acid (TBOA). Similarly, TBOA blocks visual stimulus-induced increases in intrinsic hemodynamic signal in ferret visual cortex (Schummers et al., 2008). Together these studies strongly suggest that glutamate transport contributes to neuronal activity-dependent increases in blood flow. All of these experiments used inhibition/ablation of glutamate uptake to infer a role for glutamate transport in the neurovascular response, however, none tested for a direct effect of glutamate transport activation.

The goal of the present study was to address this gap and to test a model by which glutamate uptake might contribute to neurovascular coupling (schematized in Figure 2). We used a non-endogenous glutamate transporter substrate (L-THA) to determine if direct activation of glutamate transport is sufficient to cause an increase in arteriole diameter. We show that focal application of L-THA causes increases in arteriole diameter (maximum change and AUC) that are larger than those observed after focal application of saline. We also show that this effect is abolished by co-application of the pan-inhibitor of Na^+^-dependent glutamate transporters (TFB-TBOA). This observation is consistent with the notion that direct activation of glutamate transport is sufficient to cause an increase in arteriole diameter. Substrate inhibitors of these transporters can also stimulate the release of glutamate through hetero-exchange (Bridges et al., 1999; Danbolt, 2001). Therefore, it is also possible that L-THA may be causing an increase in extracellular glutamate/aspartate which could in turn cause an increase in arteriole diameter by activating glutamate receptors. We think this is less likely for two reasons. First, there is little evidence that the effects of L-THA were altered by co-application of inhibitors of the major glutamate receptors (DNQX, AP5, MCPG). Second, as indicated above, TBOA attenuates activity induced increases in blood flow (Gurden et al., 2006; Petzold et al., 2008; Schummers et al., 2008). These investigators were concerned that TBOA might be increasing extracellular glutamate and that glutamate might be causing vasoconstriction by activating glutamate receptors. They tested either the same cocktail of receptor antagonists that were used in the present study (Petzold et al., 2008) or slightly different receptor antagonists (Schummers et al., 2008). Based on these observations, we propose that a direct effect of glutamate transport activation on arteriole diameter is the simplest explanation of these data. Petzold and colleagues came to the same conclusion, but wondered how glutamate activation might trigger an increase in arteriole diameter (for review, see Petzold and Murthy, 2011).

One possible explanation for glutamate receptors and transporters both triggering dilations is convergence upon a common signaling pathway. Petzold et al. suggested that both pathways might converge upon increases in intracellular Ca^2+^, although through an unknown mechanism (Petzold and Murthy, 2011). Several different studies have examined the potential contributions of changes in astrocytic Ca^2+^ to changes in arteriole diameter, and the results are somewhat varied (for discussions, see Biesecker and Srienc, 2015; Khakh and McCarthy, 2015; Munoz et al., 2015; Bazargani and Attwell, 2016; Lia et al., 2021). Takano and colleagues photo-uncaged Ca^2+^ in astrocyte endfeet and found that this causes an increase in blood flow *in vivo* (Takano et al., 2006). Others have linked increases in astrocytic Ca^2+^ to vasodilation, to vasoconstriction, and finally to bi-directional control vessel diameter (Zonta et al., 2003; Winship et al., 2007; Girouard et al., 2010; Gordon et al., 2011; Nizar et al., 2013; Institoris et al., 2015; Otsu et al., 2015; Biesecker et al., 2016; Lind et al., 2018). Ca^2+^ signaling is complex with multiple pathways/sources that can cause increases in cytoplasmic levels of Ca^2^. In a cell with processes, it is also possible to stimulate local increases in Ca^2+^ that do not necessarily propagate throughout the cell; this has been clearly documented in astrocyte processes (O’Donnell et al., 2016; Robinson and Jackson, 2016; Agarwal et al., 2017 for reviews, see Khakh and McCarthy, 2015; Bazargani and Attwell, 2016; Lia et al., 2021). While the contributions of Ca^2+^ to the control of neurovascular coupling have been considered controversial, it seems likely that contributions are a matter of source, magnitude, and location of the change in Ca^2+^ that governs the direction of the effect on arteriole diameter.

The levels of the glutamate transporters are very high in the nervous system and GLT-1 has been estimated at 1% of brain protein (Danbolt, 2001). Both GLT-1 and GLAST display some heterogeneity in their distribution on the astrocyte membrane; they are enriched on endfeet facing the neuropil and with lower expression on the luminal side facing the blood vessel (Chaudhry et al., 1995; Lehre et al., 1995; Langer et al., 2017). The transport of glutamate by GLT-1, GLAST, and the other members of this family is accompanied by the simultaneous movement of 3Na^+^ ions and one H^+^, resulting in local depolarization and the accumulation of Na^+^ in the astrocytes (Wadiche et al., 1995; Zerangue and Kavanaugh, 1996). Over two decades ago, several groups demonstrated that glutamate uptake causes fairly robust depolarization of astrocytes; this was demonstrated using physiologic recording in the astrocyte cell body (Bergles and Jahr, 1997; Diamond et al., 1998). In a more recent study, Langer and colleagues used a Na^+^ indicator and fluorescent imaging to demonstrate that neuronal activity or a transporter substrate (D-aspartate) cause a rapid increase Na^+^ in astrocytic endfeet; these Na^+^ signals can travel at speeds of up to 120 μm/sec (Langer et al., 2017). The Na^+^/Ca^2+^ exchangers are bi-directional transporters and can move these ions in either direction based on the membrane potential (Blaustein and Lederer, 1999; Blaustein et al., 2002). The resting potential of an astrocyte is not far from this reversal potential. Several studies have demonstrated the glutamate transport is linked to either increases in Ca^2+^ or downstream effects of glutamate transport activation using agents that selectively block the reversed operation of these Na^+^/Ca^2+^ exchangers in cell culture systems (Reyes and Parpura, 2008; Magi et al., 2013; Rojas et al., 2013; Balderas et al., 2014; Parpura et al., 2016; Rose et al., 2020). It has been suggested that these exchangers might contribute to regenerative Ca^2+^ in astrocyte processes (Brazhe et al., 2018). Schummers and colleagues demonstrated the inhibition of glutamate transport with TBOA blocks stimulus induced increases Ca *in vivo* (Schummers et al., 2008). We found that TFB-TBOA or YM244769 (an inhibitor of reversed Na^+^/Ca^2+^ exchanger) reduce basil Ca^2+^ in astrocyte processes in an organotypic slice (Jackson and Robinson, 2015). A recent study also identified a Ca^2+^ signal in radial astrocytes that was blocked by either inhibiting glutamate uptake or these Na^+^/ Ca^2+^ exchangers in the developing xenopus (Benfey et al., 2021). Together these studies strongly suggest that glutamate transport and reversed Na^+^/Ca^2+^ exchange are coupled to increased increases in Ca^2+^ in astrocytes, but these effects have not been linked to glutamate transport-induced increases in arteriole diameter. In future studies, it will be important to examine the direct effects of glutamate transport activation on Ca^2+^ in astrocyte processes. If our model is correct, one would predict that glutamate transport-induced changes in astrocyte Ca^2+^ would follow kinetics that are consistent with changes in arteriole diameter. Although not trivial, these studies can be performed by selectively expressing genetically encoded Ca^2+^ indicators in astrocytes and testing the effects of inhibitors of various sources of Ca^2+^ including the Na^+^/Ca^2+^ exchangers.

In the model, we also show two different Ca^2+^ activated processes that have been previously linked to increases arteriole diameter, nitric oxide synthase and signaling through phospholipase A2 and activation of cyclooxygenase. While we cannot rule-out the contributions of either of these potential pathways to transport-induced increases in arteriole diameter. Petzold and colleagues linked cyclo-oxygenase to mGluR-dependent dilations and provide evidence that transport-dependent dilations are independent of cyclo-oxygenase (Petzold et al., 2008). The signals downstream of glutamate transport will need further investigation.

Why couple glutamate transport to local blood flow control? Astrocytes play an important role in maintaining excitatory neurotransmission via the clearance of synaptically released glutamate. Glutamate is almost entirely cleared into astrocytes via GLAST and GLT-1 (Rothstein et al., 1994; Tanaka et al., 1997; Robinson, 1999; Danbolt, 2001). The glutamate transporters couple the movement of glutamate with the co-transport of 3Na^+^ and a H^+^ and the counter transport of a K^+^ ion (Zerangue and Kavanaugh, 1996). This is in turn coupled to activation of the Na^+^/ K^+^ ATPase and hydrolysis of ATP. Thus, glutamate transport consumes energy (Attwell and Laughlin, 2001). Glutamate uptake causes activation of the Glut1 subtype of glucose transporter in astrocytes (Loaiza et al., 2003; Porras et al., 2008) and increases in glycolysis and lactate release (Voutsinos-Porche et al., 2003). Coupling of glutamate transport to changes in local blood flow would potentially provide astrocytic access to energetic substrates necessary to maintain glutamate uptake and to support the other metabolic costs of glutamate recycling.

Like many other cells in the nervous system, astrocytes are heterogeneous (for reviews, see Farmer and Murai, 2017; Khakh and Deneen, 2019; Kohler et al., 2021). Therefore, it is possible that glutamate transport may not be coupled to changes in arteriole diameter in all brain areas, but the fact that genetic deletion or inhibition of glutamate transport attenuates activity-dependent increases in blood flow or energy delivery in different areas of the cortex, olfactory bulb, visual cortex, and sensory cortex suggests that this may be a widespread phenomenon. Although there is astrocyte heterogeneity, the levels of GLT-1 do not vary much across the forebrain (Danbolt, 2001) and are not different in at least two populations of cortical astrocytes (Miller et al., 2019).

During the analysis of changes in arteriole diameter in response to L-THA (and other substrates), we encountered some variability to the responses both in terms of overall vessel responsiveness and local responsiveness. When we explored the data visually, it is apparent that there are animals that are responders and non-responders (Figure 9), although we did not try to make any statistical inferences. Differences in the responsiveness of individual vessel segments in response to the same stimulus is also apparent (Figure 9). Most of our results for the inhibitors were not statistically conclusive, and it is possible that there was indeed no effect. However, in most cases we were intrigued by trends toward inhibition in at least two of the three pulses. It is possible that we were underpowered to detect biologically relevant effects. When designing a study of this type, the statistical power for a given sample size depends on the ratio of the change in AUC to the standard deviation of the outcome. Larger changes in AUC might be effected by optimizing concentrations of drugs, but these experiments would be time consuming. The standard deviation reflects two variance components: the between-animal variability and the within-animal variability. For example, for AUC (ln scale), the total variance in our models had an estimated value of 5.75 with a between-animal component of 1.24 and a within-animal variance of 4.51. Increasing sample sizes beyond 5-6 animals per group is probably not realistic given the logistical cost of doing this experiment. However, if the variance could be reduced, one could obtain better precision. In addition to neurovascular coupling, blood flow is maintained by a process termed “cerebral autoregulation.” This is essentially a pressure-dependent phenomenon. When blood pressure increases, vessel diameter decreases, and the opposite occurs when blood pressure decreases (for recent reviews/discussions see Filosa et al., 2015; Kim et al., 2015, 2016; Rosenegger et al., 2015). We did not control for blood pressure in this study and changes in blood pressure could increase between pulse and between animal variability. Another way to reduce variability would be to more closely target the anatomy so that the pipette was consistently in the region of an astrocyte that projects an endfoot to the area of the arteriole being imaged. While the specialized endfoot processes of astrocytes are estimated to cover 99.7% of the abluminal surface of the arteriole surface (Simard and Nedergaard, 2004; Mathiisen et al., 2010), the structural relationship of any one astrocyte to a specific segment of arteriole is likely to be highly restricted. Previous anatomic studies using confocal microscopy demonstrated diversity in the extent to which astrocytes contact vascular elements, with a single astrocyte contacting several vascular sites via many endfeet or a single vascular site receiving endfoot contact from several astrocytes (Kacem et al., 1998). Finally, as mentioned in the previous paragraph, there is clear evidence for astrocyte heterogeneity, it is possible that this heterogeneity also contributes to the variability observed in the current studies.

In conclusion, our study provided a physiologically intact system with normal blood pressure and blood flow. We wanted to adapt a method that could be used to monitor arteriole diameter and to acutely modulate glutamate transporters *in vivo*. Here, we employed two-photon *in vivo* microscopy along with acute labeling of the vasculature (arterioles) using fluorescent dyes to monitor the diameter of arterioles *in vivo*. Using an acute, partial cranial window and local application of transporter substrates and inhibitors, we were able to demonstrate that glutamate transport alone is sufficient to evoke arteriole dilation in the cortex of live mice. We created image-analysis methods, implemented in freely available code, and established a workflow to analyze the two-photon time-lapse micrographs in an automated objective manner. This workflow will greatly simplify future analyses of blood vessel diameter and contribute to a mechanistic dissection of various downstream signals (NCX activation, mitochondrial) or parallel pathways controlling NVC.

## Data Availability Statement

The raw data supporting the conclusions of this article will be made available by the authors, without undue reservation.

## Ethics Statement

The animal study was reviewed and approved by Institutional Animal Care and Use Committee at the Children’s Hospital of Philadelphia.

## Author Contributions

JJ designed and conducted the experiments, contributed to the data analysis, and the preparation of the manuscript. EK contributed to the data analysis, and preparation of figures and the manuscript. HT contributed to the experimental design, made BloodV10 & KymoHT6, and contributed to preparation of the manuscript. ML contributed to the data analysis, wrote the Python script, and contributed to the preparation of the manuscript. GC wrote the code for statistical comparisons. MP oversaw and conducted statistical analyses and wrote several sections of the manuscript. MR contributed to conceptualization of the study, to data analyses and interpretation, and to preparation of the manuscript. All authors reviewed and approved the final version of the manuscript.

## Conflict of Interest

The authors declare that the research was conducted in the absence of any commercial or financial relationships that could be construed as a potential conflict of interest.

## Publisher’s Note

All claims expressed in this article are solely those of the authors and do not necessarily represent those of their affiliated organizations, or those of the publisher, the editors and the reviewers. Any product that may be evaluated in this article, or claim that may be made by its manufacturer, is not guaranteed or endorsed by the publisher.

## Footnotes

1
www.github.com/takano2050/BloodVesselAnalysis

2
https://github.com/leemc-data-ed/blood-vessel-diameter

## References

[B1] Agarwal A. Wu P. H. Hughes E. G. Fukaya M. Tischfield M. A. Langseth A. J. (2017). Transient opening of the mitochondrial permeability transition pore induces microdomain calcium transients in astrocyte processes. *Neuron* 93 587–605 e587. 10.1016/j.neuron.2016.12.034 28132831 PMC5308886

[B2] Arriza J. L. Fairman W. A. Wadiche J. I. Murdoch G. H. Kavanaugh M. P. Amara S. G. (1994). Functional comparisons of three glutamate transporter subtypes cloned from human motor cortex. *J. Neurosci.* 14 5559–5569. 10.1523/JNEUROSCI.14-09-05559.1994 7521911 PMC6577102

[B3] Attwell D. Laughlin S. B. (2001). An energy budget for signaling in the grey matter of the brain. *J. Cereb. Blood Flow Metab.* 21 1133–1145. 10.1097/00004647-200110000-00001 11598490

[B4] Attwell D. Buchan A. M. Charpak S. Lauritzen M. Macvicar B. A. Newman E. A. (2010). Glial and neuronal control of brain blood flow. *Nature* 468 232–243. 10.1038/nature09613 21068832 PMC3206737

[B5] Balderas A. Guillem A. M. Martinez-Lozada Z. Hernandez-Kelly L. C. Aguilera J. Ortega A. (2014). GLAST/EAAT1 regulation in cultured *Bergmann glia* cells: role of the NO/cGMP signaling pathway. *Neurochem. Int.* 73 139–145. 10.1016/j.neuint.2013.10.011 24211711

[B6] Bazargani N. Attwell D. (2016). Astrocyte calcium signaling: the third wave. *Nat. Neurosci.* 19 182–189. 10.1038/nn.4201 26814587

[B7] Benfey N. J. Li V. J. Schohl A. Ruthazer E. S. (2021). Sodium-calcium exchanger mediates sensory-evoked glial calcium transients in the developing retinotectal system. *Cell Rep.* 37:109791. 10.1016/j.celrep.2021.109791 34610307

[B8] Bergles D. E. Jahr C. E. (1997). Synaptic activation of glutamate transporters in hippocampal astrocytes. *Neuron* 19 1297–1308. 10.1016/s0896-6273(00)80420-1 9427252

[B9] Biesecker K. R. Srienc A. I. (2015). The functional role of astrocyte calcium signaling in cortical blood flow regulation. *J. Neurosci.* 35 868–870. 10.1523/JNEUROSCI.4422-14.2015 25609605 PMC4300329

[B10] Biesecker K. R. Srienc A. I. Shimoda A. M. Agarwal A. Bergles D. E. Kofuji P. (2016). Glial cell calcium signaling mediates capillary regulation of blood flow in the retina. *J. Neurosci.* 36 9435–9445. 10.1523/JNEUROSCI.1782-16.2016 27605617 PMC5013190

[B11] Blaustein M. P. Lederer W. J. (1999). Sodium/calcium exchange: its physiological implications. *Physiol. Rev.* 79 763–854. 10.1152/physrev.1999.79.3.763 10390518

[B12] Blaustein M. P. Juhaszova M. Golovina V. A. Church P. J. Stanley E. F. (2002). Na/Ca exchanger and PMCA localization in neurons and astrocytes: functional implications. *Ann. N. Y. Acad. Sci.* 976 356–366. 10.1111/j.1749-6632.2002.tb04762.x 12502582

[B13] Bohn K. A. Adkins C. E. Mittapalli R. K. Terrell-Hall T. B. Mohammad A. S. Shah N. (2016). Semi-automated rapid quantification of brain vessel density utilizing fluorescent microscopy. *J. Neurosci. Methods* 270 124–131. 10.1016/j.jneumeth.2016.06.012 27321229 PMC4981522

[B14] Brazhe A. R. Verisokin A. Y. Verveyko D. V. Postnov D. E. (2018). Sodium-calcium exchanger can account for regenerative Ca(2+) entry in thin astrocyte processes. *Front. Cell Neurosci.* 12:250. 10.3389/fncel.2018.00250 30154700 PMC6102320

[B15] Bridges R. J. Kavanaugh M. P. Chamberlin A. R. (1999). A pharmacological review of competitive inhibitors and substrates of high-affinity, sodium-dependent glutamate transport in the central nervous system. *Curr. Pharm. Des.* 5 363–379. 10213800

[B16] Chaudhry F. A. Lehre K. P. Campagne M. V. L. Ottersen O. P. Danbolt N. C. Storm-Mathisen J. (1995). Glutamate transporters in glial plasma membranes: highly differentiated localizations revealed by quantitative ultrastructural immunocytochemistry. *Neuron* 15 711–720. 10.1016/0896-6273(95)90158-2 7546749

[B17] Cholet N. Pellerin L. Welker E. Lacombe P. Seylaz J. Magistretti P. (2001). Local injection of antisense oligonucleotides targeted to the glial glutamate transporter GLAST decreases the metabolic response to somatosensory activation. *J. Cereb. Blood Flow Metab.* 21 404–412. 10.1097/00004647-200104000-00009 11323526

[B18] Danbolt N. C. (2001). Glutamate uptake. *Prog. Neurobiol.* 65 1–105. 11369436 10.1016/s0301-0082(00)00067-8

[B19] Danbolt N. C. Furness D. N. Zhou Y. (2016). Neuronal vs glial glutamate uptake: resolving the conundrum. *Neurochem. Int.* 98 29–45. 10.1016/j.neuint.2016.05.009 27235987

[B20] Diamond J. S. Bergles D. E. Jahr C. E. (1998). Glutamate release monitored with astrocyte transporter currents during LTP. *Neuron* 21 425–433. 10.1016/s0896-6273(00)80551-6 9728923

[B21] Erreger K. Geballe M. T. Kristensen A. Chen P. E. Hansen K. B. Lee C. J. (2007). Subunit-specific agonist activity at NR2A-, NR2B-, NR2C-, and NR2D-containing N-methyl-D-aspartate glutamate receptors. *Mol. Pharmacol.* 72 907–920. 10.1124/mol.107.037333 17622578

[B22] Faraci F. M. Breese K. R. (1993). Nitric oxide mediates vasodilatation in response to activation of N-methyl-D-aspartate receptors in brain. *Circ. Res.* 72 476–480. 10.1161/01.res.72.2.476 8380361

[B23] Farmer W. T. Murai K. (2017). Resolving astrocyte heterogeneity in the CNS. *Front. Cell Neurosci.* 11:300. 10.3389/fncel.2017.00300 29021743 PMC5623685

[B24] Filosa J. A. Iddings J. A. (2013). Astrocyte regulation of cerebral vascular tone. *Am. J. Physiol. Heart Circ. Physiol.* 305 H609–H619. 10.1152/ajpheart.00359.2013 23792684 PMC3761330

[B25] Filosa J. A. Bonev A. D. Nelson M. T. (2004). Calcium dynamics in cortical astrocytes and arterioles during neurovascular coupling. *Circ. Res.* 95 e73–e81. 10.1161/01.RES.0000148636.60732.2e 15499024

[B26] Filosa J. A. Morrison H. W. Iddings J. A. Du W. Kim K. J. (2015). Beyond neurovascular coupling, role of astrocytes in the regulation of vascular tone. *Neuroscience* 323 96–109. 10.1016/j.neuroscience.2015.03.064 25843438 PMC4592693

[B27] Garthwaite J. (1985). Cellular uptake disguises action of L-glutamate on N-methyl-D-aspartate receptors. *Br. J. Pharmacol.* 85 297–307. 10.1111/j.1476-5381.1985.tb08860.x 2862941 PMC1916772

[B28] Girouard H. Bonev A. D. Hannah R. M. Meredith A. Aldrich R. W. Nelson M. T. (2010). Astrocytic endfoot Ca2+ and BK channels determine both arteriolar dilation and constriction. *Proc. Natl. Acad. Sci. U.S.A.* 107 3811–3816. 10.1073/pnas.0914722107 20133576 PMC2840528

[B29] Gordon G. R. Choi H. B. Rungta R. L. Ellis-Davies G. C. Macvicar B. A. (2008). Brain metabolism dictates the polarity of astrocyte control over arterioles. *Nature* 456 745–749. 10.1038/nature07525 18971930 PMC4097022

[B30] Gordon G. R. Howarth C. Macvicar B. A. (2011). Bidirectional control of arteriole diameter by astrocytes. *Exp. Physiol.* 96 393–399. 10.1113/expphysiol.2010.053132 21257665

[B31] Gurden H. (2013). Astrocytes: can they be the missing stars linking neuronal activity to neurofunctional imaging signals? *Front. Cell Neurosci.* 7:21. 10.3389/fncel.2013.00021 23476628 PMC3592263

[B32] Gurden H. Uchida N. Mainen Z. F. (2006). Sensory-evoked intrinsic optical signals in the olfactory bulb are coupled to glutamate release and uptake. *Neuron* 52 335–345. 10.1016/j.neuron.2006.07.022 17046695

[B33] Hamilton N. B. Attwell D. Hall C. N. (2010). Pericyte-mediated regulation of capillary diameter: a component of neurovascular coupling in health and disease. *Front. Neuroenerget.* 2:5. 10.3389/fnene.2010.00005 20725515 PMC2912025

[B34] Harris J. J. Jolivet R. Attwell D. (2012). Synaptic energy use and supply. *Neuron* 75 762–777. 10.1016/j.neuron.2012.08.019 22958818

[B35] Herard A. S. Dubois A. Escartin C. Tanaka K. Delzescaux T. Hantraye P. (2005). Decreased metabolic response to visual stimulation in the superior colliculus of mice lacking the glial glutamate transporter GLT-1. *Eur. J. Neurosci.* 22 1807–1811. 10.1111/j.1460-9568.2005.04346.x 16197522

[B36] Herman M. A. Jahr C. E. (2007). Extracellular glutamate concentration in hippocampal slice. *J. Neurosci.* 27 9736–9741. 10.1523/JNEUROSCI.3009-07.2007 17804634 PMC2670936

[B37] Hertz L. Peng L. Dienel G. A. (2007). Energy metabolism in astrocytes: high rate of oxidative metabolism and spatiotemporal dependence on glycolysis/glycogenolysis. *J. Cereb. Blood Flow Metab.* 27 219–249. 10.1038/sj.jcbfm.9600343 16835632

[B38] Hill R. A. Tong L. Yuan P. Murikinati S. Gupta S. Grutzendler J. (2015). Regional blood flow in the normal and ischemic brain is controlled by arteriolar smooth muscle cell contractility and not by capillary pericytes. *Neuron* 87 95–110. 10.1016/j.neuron.2015.06.001 26119027 PMC4487786

[B39] Hillman E. M. (2014). Coupling mechanism and significance of the BOLD signal: a status report. *Annu. Rev. Neurosci.* 37 161–181. 10.1146/annurev-neuro-071013-014111 25032494 PMC4147398

[B40] Hinzman J. M. Andaluz N. Shutter L. A. Okonkwo D. O. Pahl C. Strong A. J. (2014). Inverse neurovascular coupling to cortical spreading depolarizations in severe brain trauma. *Brain* 137 2960–2972. 10.1093/brain/awu241 25154387

[B41] Howarth C. (2014). The contribution of astrocytes to the regulation of cerebral blood flow. *Front. Neurosci.* 8:103. 10.3389/fnins.2014.00103 24847203 PMC4023041

[B42] Howarth C. Gleeson P. Attwell D. (2012). Updated energy budgets for neural computation in the neocortex and cerebellum. *J. Cereb. Blood Flow Metab.* 32 1222–1232. 10.1038/jcbfm.2012.35 22434069 PMC3390818

[B43] Iadecola C. Nedergaard M. (2007). Glial regulation of the cerebral microvasculature. *Nat. Neurosci.* 10 1369–1376. 10.1038/nn2003 17965657

[B44] Institoris A. Rosenegger D. G. Gordon G. R. (2015). Arteriole dilation to synaptic activation that is sub-threshold to astrocyte endfoot Ca2+ transients. *J. Cereb. Blood Flow Metab.* 35 1411–1415. 10.1038/jcbfm.2015.141 26126870 PMC4640329

[B45] Jackman K. Iadecola C. (2015). Neurovascular regulation in the ischemic brain. *Antioxid. Redox. Signal.* 22 149–160. 10.1089/ars.2013.5669 24328757 PMC4281847

[B46] Jackson J. G. Robinson M. B. (2015). Reciprocal regulation of mitochondrial dynamics and calcium signaling in astrocyte processes. *J. Neurosci.* 35 15199–15213. 10.1523/JNEUROSCI.2049-15.2015 26558789 PMC4642244

[B47] Kacem K. Lacombe P. Seylaz J. Bonvento G. (1998). Structural organization of the perivascular astrocyte endfeet and their relationship with the endothelial glucose transporter: a confocal microscopy study. *Glia* 23 1–10. 10.1002/(sici)1098-1136(199805)23:1<1::aid-glia1>3.0.co;2-b 9562180

[B48] Khakh B. S. Deneen B. (2019). The emerging nature of astrocyte diversity. *Annu. Rev. Neurosci.* 42 187–207. 10.1146/annurev-neuro-070918-050443 31283899

[B49] Khakh B. S. McCarthy K. D. (2015). Astrocyte calcium signaling: from observations to functions and the challenges therein. *Cold Spring Harb. Perspect. Biol.* 7:a020404. 10.1101/cshperspect.a020404 25605709 PMC4382738

[B50] Kim K. J. Iddings J. A. Stern J. E. Blanco V. M. Croom D. Kirov S. A. (2015). Astrocyte contributions to flow/pressure-evoked parenchymal arteriole vasoconstriction. *J. Neurosci.* 35 8245–8257. 10.1523/JNEUROSCI.4486-14.2015 26019339 PMC4444545

[B51] Kim K. J. Ramiro Diaz J. Iddings J. A. Filosa J. A. (2016). Vasculo-neuronal coupling: retrograde vascular communication to brain neurons. *J. Neurosci.* 36 12624–12639. 10.1523/JNEUROSCI.1300-16.2016 27821575 PMC5157107

[B52] Kitaura H. Uozumi N. Tohmi M. Yamazaki M. Sakimura K. Kudoh M. (2007). Roles of nitric oxide as a vasodilator in neurovascular coupling of mouse somatosensory cortex. *Neurosci. Res.* 59 160–171. 10.1016/j.neures.2007.06.1469 17655958

[B53] Kirischuk S. Kettenmann H. Verkhratsky A. (1997). Na^+^/Ca2^+^ exchanger modulates kainate-triggered Ca2^+^ signaling in Bergmann glial cells *in situ*. *FASEB J.* 11 566–572. 10.1096/fasebj.11.7.9212080 9212080

[B54] Koehler R. C. Roman R. J. Harder D. R. (2009). Astrocytes and the regulation of cerebral blood flow. *Trends Neurosci.* 32 160–169. 19162338 10.1016/j.tins.2008.11.005

[B55] Kohler S. Winkler U. Hirrlinger J. (2021). Heterogeneity of astrocytes in grey and white matter. *Neurochem. Res.* 46 3–14. 10.1007/s11064-019-02926-x 31797158

[B56] Kugler P. Drenckhahn D. (1996). Astrocytes and Bergmann glia as an important site of nitric oxide synthase I. *Glia* 16 165–173. 10.1002/(SICI)1098-1136(199602)16:2&lt;165::AID-GLIA8&gt;3.0.CO;2-2 8929903

[B57] Langer J. Gerkau N. J. Derouiche A. Kleinhans C. Moshrefi-Ravasdjani B. Fredrich M. (2017). Rapid sodium signaling couples glutamate uptake to breakdown of ATP in perivascular astrocyte endfeet. *Glia* 65 293–308. 10.1002/glia.23092 27785828

[B58] Leeuwis A. E. Benedictus M. R. Kuijer J. P. A. Binnewijzend M. A. A. Hooghiemstra A. M. Verfaillie S. C. J. (2017). Lower cerebral blood flow is associated with impairment in multiple cognitive domains in Alzheimer’s disease. *Alzheimers Dement.* 13 531–540. 10.1016/j.jalz.2016.08.013 27693109

[B59] Leeuwis A. E. Smith L. A. Melbourne A. Hughes A. D. Richards M. Prins N. D. (2018). Cerebral blood flow and cognitive functioning in a community-based, multi-ethnic cohort: the sabre study. *Front. Aging Neurosci.* 10:279. 10.3389/fnagi.2018.00279 30279656 PMC6154257

[B60] Lehre K. P. Levy L. M. Ottersen O. P. Storm-Mathisen J. Danbolt N. C. (1995). Differential expression of two glial glutamate transporters in the rat brain: quantitative and immunocytochemical observations. *J. Neurosci.* 15 1835–1853. 10.1523/JNEUROSCI.15-03-01835.1995 7891138 PMC6578153

[B61] Lia A. Henriques V. J. Zonta M. Chiavegato A. Carmignoto G. Gomez-Gonzalo M. (2021). Calcium signals in astrocyte microdomains, a decade of great advances. *Front. Cell Neurosci.* 15:673433. 10.3389/fncel.2021.673433 34163329 PMC8216559

[B62] Lind B. L. Jessen S. B. Lonstrup M. Josephine C. Bonvento G. Lauritzen M. (2018). Fast Ca(2+) responses in astrocyte end-feet and neurovascular coupling in mice. *Glia* 66 348–358. 10.1002/glia.23246 29058353

[B63] Loaiza A. Porras O. H. Barros L. F. (2003). Glutamate triggers rapid glucose transport stimulation in astrocytes as evidenced by real-time confocal microscopy. *J. Neurosci.* 23 7337–7342. 10.1523/JNEUROSCI.23-19-07337.2003 12917367 PMC6740433

[B64] Luke S. G. (2017). Evaluating significance in linear mixed-effects models in R. *Behav. Res. Methods* 49 1494–1502. 10.3758/s13428-016-0809-y 27620283

[B65] Magi S. Arcangeli S. Castaldo P. Nasti A. A. Berrino L. Piegari E. (2013). Glutamate-induced ATP synthesis: relationship between plasma membrane Na+/Ca2+ exchanger and excitatory amino acid transporters in brain and heart cell models. *Mol. Pharmacol.* 84 603–614. 10.1124/mol.113.087775 23913256

[B66] Martin C. Houitte D. Guillermier M. Petit F. Bonvento G. Gurden H. (2012). Alteration of sensory-evoked metabolic and oscillatory activities in the olfactory bulb of GLAST-deficient mice. *Front. Neural Circuits* 6:1. 10.3389/fncir.2012.00001 22291618 PMC3265768

[B67] Masamoto K. Unekawa M. Watanabe T. Toriumi H. Takuwa H. Kawaguchi H. (2015). Unveiling astrocytic control of cerebral blood flow with optogenetics. *Sci. Rep.* 5:11455. 10.1038/srep11455 26076820 PMC4468581

[B68] Mateo C. Knutsen P. M. Tsai P. S. Shih A. Y. Kleinfeld D. (2017). Entrainment of arteriole vasomotor fluctuations by neural activity is a basis of blood-oxygenation-level-dependent “resting-state” connectivity. *Neuron* 96 936–948.e933. 10.1016/j.neuron.2017.10.012 29107517 PMC5851777

[B69] Mathiisen T. M. Lehre K. P. Danbolt N. C. Ottersen O. P. (2010). The perivascular astroglial sheath provides a complete covering of the brain microvessels: an electron microscopic 3D reconstruction. *Glia* 58 1094–1103. 10.1002/glia.20990 20468051

[B70] Miller S. J. Philips T. Kim N. Dastgheyb R. Chen Z. Hsieh Y. C. (2019). Molecularly defined cortical astroglia subpopulation modulates neurons via secretion of Norrin. *Nat. Neurosci.* 22 741–752. 10.1038/s41593-019-0366-7 30936556 PMC6551209

[B71] Minelli A. Castaldo P. Gobbi P. Salucci S. Magi S. Amoroso S. (2007). Cellular and subcellular localization of Na+-Ca2+ exchanger protein isoforms, NCX1, NCX2, and NCX3 in cerebral cortex and hippocampus of adult rat. *Cell Calcium* 41 221–234. 10.1016/j.ceca.2006.06.004 16914199

[B72] Mishra A. Reynolds J. P. Chen Y. Gourine A. V. Rusakov D. A. Attwell D. (2016). Astrocytes mediate neurovascular signaling to capillary pericytes but not to arterioles. *Nat. Neurosci.* 19 1619–1627. 27775719 10.1038/nn.4428 PMC5131849

[B73] Mulligan S. J. MacVicar B. A. (2004). Calcium transients in astrocyte endfeet cause cerebrovascular constrictions. *Nature* 431 195–199. 10.1038/nature02827 15356633

[B74] Munoz M. F. Puebla M. Figueroa X. F. (2015). Control of the neurovascular coupling by nitric oxide-dependent regulation of astrocytic Ca(2+) signaling. *Front. Cell Neurosci.* 9:59. 10.3389/fncel.2015.00059 25805969 PMC4354411

[B75] Navarrete M. Perea G. Fernandez De Sevilla D. Gomez-Gonzalo M. Nunez A. Martin E. D. (2012). Astrocytes mediate in vivo cholinergic-induced synaptic plasticity. *PLoS Biol.* 10:e1001259. 10.1371/journal.pbio.1001259 22347811 PMC3279365

[B76] Nimmerjahn A. Kirchhoff F. Kerr J. N. Helmchen F. (2004). Sulforhodamine 101 as a specific marker of astroglia in the neocortex in vivo. *Nat. Methods* 1 31–37. 10.1038/nmeth706 15782150

[B77] Nizar K. Uhlirova H. Tian P. Saisan P. A. Cheng Q. Reznichenko L. (2013). In vivo stimulus-induced vasodilation occurs without IP3 receptor activation and may precede astrocytic calcium increase. *J. Neurosci.* 33 8411–8422. 10.1523/JNEUROSCI.3285-12.2013 23658179 PMC3712855

[B78] Obel L. F. Muller M. S. Walls A. B. Sickmann H. M. Bak L. K. Waagepetersen H. S. (2012). Brain glycogen-new perspectives on its metabolic function and regulation at the subcellular level. *Front. Neuroenerget.* 4:3. 10.3389/fnene.2012.00003 22403540 PMC3291878

[B79] O’Donnell J. C. Jackson J. G. Robinson M. B. (2016). Transient oxygen/glucose deprivation causes a delayed loss of mitochondria and increases spontaneous calcium signaling in astrocytic processes. *J. Neurosci.* 36 7109–7127. 10.1523/JNEUROSCI.4518-15.2016 27383588 PMC4938859

[B80] Otsu Y. Couchman K. Lyons D. G. Collot M. Agarwal A. Mallet J. M. (2015). Calcium dynamics in astrocyte processes during neurovascular coupling. *Nat. Neurosci.* 18 210–218. 10.1038/nn.3906 25531572 PMC4651918

[B81] Pappalardo L. W. Samad O. A. Black J. A. Waxman S. G. (2014). Voltage-gated sodium channel Nav 1.5 contributes to astrogliosis in an in vitro model of glial injury via reverse Na+ /Ca2+ exchange. *Glia* 62 1162–1175. 10.1002/glia.22671 24740847 PMC4060891

[B82] Parpura V. Sekler I. Fern R. (2016). Plasmalemmal and mitochondrial Na(+) -Ca(2+) exchange in neuroglia. *Glia* 64 1646–1654. 10.1002/glia.22975 27143128

[B83] Petzold G. C. Albeanu D. F. Sato T. F. Murthy V. N. (2008). Coupling of neural activity to blood flow in olfactory glomeruli is mediated by astrocytic pathways. *Neuron* 58 897–910. 10.1016/j.neuron.2008.04.029 18579080 PMC2922004

[B84] Petzold G. C. Murthy V. N. (2011). Role of astrocytes in neurovascular coupling. *Neuron* 71 782–797. 10.1016/j.neuron.2011.08.009 21903073

[B85] Porras O. H. Ruminot I. Loaiza A. Barros L. F. (2008). Na(+)-Ca(2+) cosignaling in the stimulation of the glucose transporter GLUT1 in cultured astrocytes. *Glia* 56 59–68. 10.1002/glia.20589 17924581

[B86] Porter J. T. McCarthy K. D. (1996). Hippocampal astrocytes in situ respond to glutamate released from synaptic terminals. *J. Neurosci.* 16 5073–5081. 10.1523/JNEUROSCI.16-16-05073.1996 8756437 PMC6579292

[B87] Putt M. E. (2021). Assessing risk factors with information beyond P value thresholds: statistical significance does not equal clinical importance. *Cancer* 127 1180–1185. 10.1002/cncr.33369 33351186

[B88] R Core Team (2021). *R: A Language and Environment for Statistical Computing.* Vienna: R Foundation for Statistical Computing.

[B89] Raichle M. E. Mintun M. A. (2006). Brain work and brain imaging. *Annu. Rev. Neurosci.* 29 449–476. 10.1146/annurev.neuro.29.051605.112819 16776593

[B90] Reyes R. C. Parpura V. (2008). Mitochondria modulate Ca2+-dependent glutamate release from rat cortical astrocytes. *J. Neurosci.* 28 9682–9691. 10.1523/JNEUROSCI.3484-08.2008 18815254 PMC2614891

[B122] Reyes R. C. Verkhratsky A. Parpura V. (2012). Plasmalemmal Na^+^/Ca^2 +^ exchanger modulates Ca^2 +^-dependent exocytotic release of glutamate from rat cortical astrocytes. *ASN Neuro.* 4:e00075. 10.1042/AN20110059 22268447 PMC3284767

[B91] Robinson M. B. (1999). The family of sodium-dependent glutamate transporters: a focus on the GLT-1/EAAT2 subtype. *Neurochem. Int.* 33 479–491. 10.1016/s0197-0186(98)00055-2 10098717

[B92] Robinson M. B. Jackson J. G. (2016). Astroglial glutamate transporters coordinate excitatory signaling and brain energetics. *Neurochem. Int.* 98 56–71. 10.1016/j.neuint.2016.03.014 27013346 PMC4969184

[B93] Rojas H. Colina C. Ramos M. Benaim G. Jaffe E. Caputo C. (2013). Sodium-calcium exchanger modulates the L-glutamate Ca(i) (2+) signalling in type-1 cerebellar astrocytes. *Adv. Exp. Med. Biol.* 961 267–274. 10.1007/978-1-4614-4756-6_22 23224886

[B94] Rose C. R. Ziemens D. Verkhratsky A. (2020). On the special role of NCX in astrocytes: translating Na(+)-transients into intracellular Ca(2+) signals. *Cell Calcium* 86:102154. 10.1016/j.ceca.2019.102154 31901681

[B95] Rosenegger D. G. Tran C. H. Wamsteeker Cusulin J. I. Gordon G. R. (2015). Tonic local brain blood flow control by astrocytes independent of phasic neurovascular coupling. *J. Neurosci.* 35 13463–13474. 10.1523/JNEUROSCI.1780-15.2015 26424891 PMC6605474

[B96] Rothstein J. D. Martin L. Levey A. I. Dykes-Hoberg M. Jin L. Wu D. (1994). Localization of neuronal and glial glutamate transporters. *Neuron* 13 713–725. 10.1016/0896-6273(94)90038-8 7917301

[B97] Sato T. R. Gray N. W. Mainen Z. F. Svoboda K. (2007). The functional microarchitecture of the mouse barrel cortex. *PLoS Biol.* 5:e189. 10.1371/journal.pbio.0050189 17622195 PMC1914403

[B98] Schummers J. Yu H. Sur M. (2008). Tuned responses of astrocytes and their influence on hemodynamic signals in the visual cortex. *Science* 320 1638–1643. 10.1126/science.1156120 18566287

[B99] Shen Z. Lu Z. Chhatbar P. Y. O’herron P. Kara P. (2012). An artery-specific fluorescent dye for studying neurovascular coupling. *Nat. Methods* 9 273–276. 10.1038/nmeth.1857 22266543 PMC3392962

[B100] Shi Y. Liu X. Gebremedhin D. Falck J. R. Harder D. R. Koehler R. C. (2008). Interaction of mechanisms involving epoxyeicosatrienoic acids, adenosine receptors, and metabotropic glutamate receptors in neurovascular coupling in rat whisker barrel cortex. *J. Cereb. Blood Flow Metab.* 28 111–125. 10.1038/sj.jcbfm.9600511 17519974 PMC2204069

[B101] Shimamoto K. Sakai R. Takaoka K. Yumoto N. Nakajima T. Amara S. G. (2004). Characterization of novel L-threo-beta-benzyloxyaspartate derivatives, potent blockers of the glutamate transporters. *Mol. Pharmacol.* 65 1008–1015. 10.1124/mol.65.4.1008 15044631

[B102] Shulman R. G. Rothman D. L. Behar K. L. Hyder F. (2004). Energetic basis of brain activity: implications for neuroimaging. *Trends Neurosci.* 27 489–495. 10.1016/j.tins.2004.06.005 15271497

[B103] Simard M. Nedergaard M. (2004). The neurobiology of glia in the context of water and ion homeostasis. *Neuroscience* 129 877–896. 10.1016/j.neuroscience.2004.09.053 15561405

[B104] Stobart J. L. Anderson C. M. (2013). Multifunctional role of astrocytes as gatekeepers of neuronal energy supply. *Front. Cell Neurosci.* 7:38. 10.3389/fncel.2013.00038 23596393 PMC3622037

[B105] Sun W. Mcconnell E. Pare J. F. Xu Q. Chen M. Peng W. (2013). Glutamate-dependent neuroglial calcium signaling differs between young and adult brain. *Science* 339 197–200. 10.1126/science.1226740 23307741 PMC3569008

[B106] Takano T. Tian G. F. Peng W. Lou N. Libionka W. Han X. (2006). Astrocyte-mediated control of cerebral blood flow. *Nat. Neurosci.* 9 260–267. 10.1038/nn1623 16388306

[B107] Tanaka K. Watase K. Manabe T. Yamada K. Watanabe M. Takahashi K. (1997). Epilepsy and exacerbation of brain injury in mice lacking the glutamate transporter GLT-1. *Science* 276 1699–1702. 10.1126/science.276.5319.1699 9180080

[B108] Toda N. Ayajiki K. Okamura T. (2009). Cerebral blood flow regulation by nitric oxide: recent advances. *Pharmacol. Rev.* 61 62–97. 10.1124/pr.108.000547 19293146

[B109] Togashi H. Sasaki M. Frohman E. Taira E. Ratan R. R. Dawson T. M. (1997). Neuronal (type I) nitric oxide synthase regulates nuclear factor kappaB activity and immunologic (type II) nitric oxide synthase expression. *Proc. Natl. Acad. Sci. U.S.A.* 94 2676–2680. 10.1073/pnas.94.6.2676 9122255 PMC20148

[B110] Tran C. H. Gordon G. R. (2015). Acute two-photon imaging of the neurovascular unit in the cortex of active mice. *Front. Cell Neurosci.* 9:11. 10.3389/fncel.2015.00011 25698926 PMC4318346

[B111] Uhlirova H. Kilic K. Tian P. Thunemann M. Desjardins M. Saisan P. A. (2016). Cell type specificity of neurovascular coupling in cerebral cortex. *Elife* 5:e14315. 10.7554/eLife.14315 27244241 PMC4933561

[B112] Voutsinos-Porche B. Bonvento G. Tanaka K. Steiner P. Welker E. Chatton J. Y. (2003). Glial glutamate transporters mediate a functional metabolic crosstalk between neurons and astrocytes in the mouse developing cortex. *Neuron* 37 275–286. 10.1016/s0896-6273(02)01170-4 12546822

[B113] Wadiche J. I. Arriza J. L. Amara S. G. Kavanaugh M. P. (1995). Kinetics of a human glutamate transporter. *Neuron* 14 1019–1027. 10.1016/0896-6273(95)90340-2 7748550

[B114] Wang X. Lou N. Xu Q. Tian G. F. Peng W. G. Han X. (2006). Astrocytic Ca2+ signaling evoked by sensory stimulation in vivo. *Nat. Neurosci.* 9 816–823. 10.1038/nn1703 16699507

[B115] Weber B. Barros L. F. (2015). The astrocyte: powerhouse and recycling center. *Cold Spring Harb. Perspect. Biol.* 7:a020396. 10.1101/cshperspect.a020396 25680832 PMC4665076

[B116] Winship I. R. Plaa N. Murphy T. H. (2007). Rapid astrocyte calcium signals correlate with neuronal activity and onset of the hemodynamic response in vivo. *J. Neurosci.* 27 6268–6272. 10.1523/JNEUROSCI.4801-06.2007 17554000 PMC6672142

[B117] Yang G. Zhang Y. Ross M. E. Iadecola C. (2003). Attenuation of activity-induced increases in cerebellar blood flow in mice lacking neuronal nitric oxide synthase. *Am. J. Physiol. Heart Circ. Physiol.* 285 H298–H304. 10.1152/ajpheart.00043.2003 12623792

[B118] Zerangue N. Kavanaugh M. P. (1996). Flux coupling in a neuronal glutamate transporter. *Nature* 383 634–637. 10.1038/383634a0 8857541

[B119] Zhang H. Q. Fast W. Marletta M. A. Martasek P. Silverman R. B. (1997). Potent and selective inhibition of neuronal nitric oxide synthase by N omega-propyl-L-arginine. *J. Med. Chem.* 40 3869–3870. 10.1021/jm970550g 9397167

[B120] Zhang Y. Chen K. Sloan S. A. Bennett M. L. Scholze A. R. O’keeffe S. (2014). An RNA-sequencing transcriptome and splicing database of glia, neurons, and vascular cells of the cerebral cortex. *J. Neurosci.* 34 11929–11947. 10.1523/JNEUROSCI.1860-14.2014 25186741 PMC4152602

[B121] Zonta M. Angulo M. C. Gobbo S. Rosengarten B. Hossmann K. A. Pozzan T. (2003). Neuron-to-astrocyte signaling is central to the dynamic control of brain microcirculation. *Nat. Neurosci.* 6 43–50. 10.1038/nn980 12469126

